# The Genome of *Plasmodium gonderi*: Insights into the Evolution of Human Malaria Parasites

**DOI:** 10.1093/gbe/evae027

**Published:** 2024-02-20

**Authors:** Axl S Cepeda, Beatriz Mello, M Andreína Pacheco, Zunping Luo, Steven A Sullivan, Jane M Carlton, Ananias A Escalante

**Affiliations:** Biology Department/Institute of Genomics and Evolutionary Medicine (iGEM), Temple University, Philadelphia, PA 19122-1801, USA; Departamento de Genética, Universidade Federal do Rio de Janeiro, Rio de Janeiro, Brazil; Biology Department/Institute of Genomics and Evolutionary Medicine (iGEM), Temple University, Philadelphia, PA 19122-1801, USA; Center for Genomics & Systems Biology, Department of Biology, New York University, New York, NY 10003, USA; Center for Genomics & Systems Biology, Department of Biology, New York University, New York, NY 10003, USA; Center for Genomics & Systems Biology, Department of Biology, New York University, New York, NY 10003, USA; Biology Department/Institute of Genomics and Evolutionary Medicine (iGEM), Temple University, Philadelphia, PA 19122-1801, USA

**Keywords:** phylogenomics, GC content, *Plasmodium vivax*, *Plasmodium falciparum*, molecular clock, synteny

## Abstract

*Plasmodium* species causing malaria in humans are not monophyletic, sharing common ancestors with nonhuman primate parasites. *Plasmodium gonderi* is one of the few known *Plasmodium* species infecting African old-world monkeys that are not found in apes. This study reports a de novo assembled *P. gonderi* genome with complete chromosomes. The *P. gonderi* genome shares codon usage, syntenic blocks, and other characteristics with the human parasites *Plasmodium ovale s.l.* and *Plasmodium malariae*, also of African origin, and the human parasite *Plasmodium vivax* and species found in nonhuman primates from Southeast Asia. Using phylogenetically aware methods, newly identified syntenic blocks were found enriched with conserved metabolic genes. Regions outside those blocks harbored genes encoding proteins involved in the vertebrate host-*Plasmodium* relationship undergoing faster evolution. Such genome architecture may have facilitated colonizing vertebrate hosts. Phylogenomic analyses estimated the common ancestor between *P. vivax* and an African ape parasite *P. vivax-*like, within the Asian nonhuman primates parasites clade. Time estimates incorporating *P. gonderi* placed the *P. vivax* and *P. vivax-*like common ancestor in the late Pleistocene, a time of active migration of hominids between Africa and Asia. Thus, phylogenomic and time-tree analyses are consistent with an Asian origin for *P. vivax* and an introduction of *P. vivax-*like into Africa. Unlike other studies, time estimates for the clade with *Plasmodium falciparum*, the most lethal human malaria parasite, coincide with their host species radiation, African hominids. Overall, the newly assembled genome presented here has the quality to support comparative genomic investigations in *Plasmodium*.

**Significance**

The *Plasmodium* causing human malaria originated from species found in nonhuman primates. Here, a new *Plasmodium gonderi* genome assembly is presented; this parasite is related to *Plasmodium vivax,* the most prevalent human parasite outside Africa. In addition to informing about the evolution of *Plasmodium* genome fissures, such as synteny and codon usage, phylogenomic analyses and time estimates provide scenarios for the origins of *Plasmodium* species infecting humans. *Plasmodium vivax's* most recent common ancestor was found among nonhuman primate parasites from Southeast Asia. The species related to *Plasmodium falciparum*, the agent causing the most severe human malaria, have a complex history with host switches but not necessarily among extant ape species, as these parasites could be as old as African apes.

## Introduction

The genus *Plasmodium* is a diverse taxon of vector-borne parasitic protozoa, some of which cause malaria in their vertebrate hosts, including humans (Garnham 1966; Coatney et al. 1971; Telford 2009; Valkiūnas and Iezhova 2018; Pacheco and Escalante 2023). The 5 species that typically infect humans do not form a monophyletic group (Escalante et al. 2022). Each shares its most recent common ancestors with different *Plasmodium* species found in nonhuman primates. Such distinct human parasites’ evolutionary histories translate into differences in phenotypic traits and clinical manifestations of the disease, requiring different therapeutics and control interventions (Garnham 1966; Coatney et al. 1971; Escalante et al. 2022). As a result, understanding the origins and biology of human malaria parasites has propelled genomic studies on their related species infecting nonhuman primates (Tachibana et al. 2012; Chien et al. 2016; Pasini et al. 2017; Rutledge et al. 2017; Lapp et al. 2018; Otto et al. 2018).

Taxonomists early recognized differences among *Plasmodium* species found in primates (Garnham 1966; Coatney et al. 1971), creating subgenera. E.g., *Plasmodium falciparum*, the agent of the most severe form of human malaria, was separated from the other human parasites in the subgenus *Laverania* (Garnham 1966; Coatney et al. 1971; Sinden et al. 1978). Nowadays, it is known that *P. falciparum* is part of a diverse clade of parasites infecting African apes, sometimes referred to as *Laverania* following the classical taxonomy (Ollomo et al. 2009; Krief et al. 2010; Liu et al. 2010; Pacheco et al. 2013; Liu et al. 2017). Given the interest in *P. falciparum*, most species in this clade have reference genomes (Otto et al. 2018).

The other parasites that primarily infect humans, *Plasmodium ovale s.l., Plasmodium malariae,* and *P. vivax,* were placed in a subgenus called *Plasmodium*, together with species found in catarrhine monkeys (Garnham 1966; Coatney et al. 1971). Although such a subgenus is likely paraphyletic (Pacheco et al. 2011, 2022), evidence suggests a clade of primate parasites with all nonfalciparum-related *Plasmodium* species, including species found in lemurs (Pacheco et al. 2018, 2022). Still, species infecting humans within this putative primate parasite clade do not form a monophyletic group.

Evidence shows that species infecting African apes share common ancestors with *P. malariae* and *P. ovale s.l.* (Duval et al. 2009; Rutledge et al. 2017; Mapua et al. 2018; Fuehrer et al. 2022; Plenderleith et al. 2022). Although *P. malariae* undergoes anthropozoonotic cycles between humans and platyrrhine primates in South America (Bajic et al. 2022; Fuehrer et al. 2022), its origin has been established in Africa (Rutledge et al. 2017; Plenderleith et al. 2022). Unlike other human parasites, the origin of *P. vivax*, the most common human malaria agent outside Africa, has been debated. *Plasmodium vivax* shares a common ancestor with parasite species from African and Southeast Asian nonhuman primates. These parasites exhibit extraordinary phenotypic diversity (Garnham 1966; Coatney et al. 1971) and will be further referred to as the “vivax-clade.” Also, within this vivax-clade are lineages related to *P. vivax* but infecting African apes. Such species are loosely called *P. vivax-*like, and their complex evolutionary history has driven the discussion about the origin of *P. vivax* as a human parasite (Prugnolle et al. 2013; Liu et al. 2014; Daron et al. 2021).

In addition to *P. vivax* and *P. vivax-*like, there are genomic data on some vivax-clade species found in Southeast Asia nonhuman primates as they have been used as models to understand malaria in humans, and some cause zoonotic malaria, such as *Plasmodium knowlesi* (Coatney et al. 1971; Cox-Sing et al. 2008; Tachibana et al. 2012; Pasini et al. 2017; Lapp et al. 2018; Galinski 2022). However, a complete picture regarding the origin of *P. vivax* requires understanding its phylogenetic relationships with the species infecting nonhuman primates in Southeast Asia and those found in African monkeys (Coatney et al. 1971; Poirriez et al. 1995), such as *Plasmodium gonderi*.

*Plasmodium gonderi* was first found by Gonder and von Berenberg-Cossler in 1908, infecting mangabeys (Coatney et al. 1971; Poirriez et al. 1995), and then described by Sinton and Mulligan in 1933. This parasite has also been found infecting drills (*Mandrillus leucophaeus*; Garnham et al. 1958) and mandrills (*Mandrillus sphinx*, Charpentier et al. 2019). Based on its biological traits, *P. gonderi* was considered “related” to nonhuman parasites in Southeast Asia and *P. vivax* (Coatney et al. 1971), an assumption confirmed by molecular phylogenetic studies using a single locus or multiple loci (Escalante et al. 1998; Leclerc et al. 2004; Escalante et al. 2005; Hayakawa et al. 2008; Nishimoto et al. 2008; Pacheco et al. 2018). However, the relative position of *P. gonderi* in the *Plasmodium* phylogeny has been controversial (Leclerc et al. 2004; Escalante et al. 2005; Nishimoto et al. 2008; Pacheco et al. 2018, Pacheco et al. 2020; Sharp et al. 2020; Cepeda et al. 2021; Escalante et al. 2022; Arisue et al. 2022). There is a debate about whether *P. gonderi* places the most recent common ancestor of *P. vivax* and *P. vivax-*like outside the clade of nonhuman primate parasites from Southeast Asia (Escalante et al. 2005; Pacheco et al. 2018; Arisue et al. 2019; Pacheco et al. 2020; Sharp et al. 2020; Cepeda et al. 2021; Arisue et al. 2022; Escalante et al. 2022; Pacheco et al. 2022; Pacheco and Escalante 2023). Such evidence has been interpreted as indicative of an African origin for the lineage leading to *P. vivax*. Beyond such controversies surrounding the origin of *P. vivax*, *P. gonderi* is one of the few known *Plasmodium* found in African catarrhine primates that are not apes. Thus, a good-quality genome for this species is essential for comparative studies in *Plasmodium*.

A *P. gonderi* isolate (Garnham strain, ATCC 30045) has been maintained (Coatney et al. 1971; Collins and Contacos 1980), facilitating a first draft genome (Honma et al. 2017). Although valuable (Sharp et al. 2020; Arisue et al. 2022; Escalante et al. 2022), it lacked the quality to assemble complete chromosomes without a reference, and annotating gene families was also challenging. A chromosome-level de novo assembled genome of *P. gonderi* ATCC 30045 is presented after incorporating new sequence data. This investigation offers new insights into the evolution of malaria parasites in primates, including the origins of those species infecting humans.

### Results and Discussion

#### Assembly of *Plasmodium gonderi* Genome

The reference genome presented here will be referred to as *P. gonderi* v2 (supplementary fig. S1, Supplementary Material online). Its assembly statistics were compared with the previous draft genome, *P. gonderi* v1 (Honma et al. 2017), and genomes from other *Plasmodium* species (supplementary table S1, Supplementary Material online). The average coverage of *P. gonderi* v2 was 1270 × (min = 729 × and max = 4022 × ; supplementary fig. S1, Supplementary Material online), an improvement over *P. gonderi* v1, where the average coverage was 250 × (Honma et al. 2017). As a result, *P. gonderi* v2 reduced the number of scaffolds to 72 (compared to 743 for *P. gonderi* v1) and an ambiguous nucleotide ratio of 0.86 (compared to 283.03 for *P. gonderi* v1; supplementary table S1, Supplementary Material online). The N50 of *P. gonderi* v2 is 2,125,246 versus 1,639,955 in the previous version (supplementary table S1, Supplementary Material online). Each of the *P. gonderi* v2 largest 14 scaffolds represented a chromosome, while 56 smaller scaffolds could not be assigned to specific chromosomes. Two scaffolds contain the mitochondrial and apicoplast genomes. In particular, a 173 kb scaffold contained at least 53 copies of the linear mitochondrial genome (mtDNA) (sequence ID: Pgonderi_v2_iGEM_MIT), common in *Plasmodium* spp. (Vaidya et al. 1989) and a 34.5 kb scaffold contained the genome of the apicoplast, a nonphotosynthetic plastid-like organelle homologous to the photosynthetic plastids found in related protists (Alveolate, a superphylum that includes dinoflagellates) (Wilson et al. 1996) (Pgonderi_v2_iGEM_API). The organelle sequences were identical in both *P. gonderi* assembly versions.

*Plasmodium gonderi* v2 reached metrics of other high-quality genome assemblies, such as *Plasmodium cynomolgi* (Pasini et al. 2017) and *P. vivax* (Auburn et al. 2016; supplementary table S1, Supplementary Material online). As a result, there is an impact on the structural annotation, resulting in 6,412 CoDing sequences (CDSs) (399 more than the previous version; supplementary table S1, Supplementary Material online), excluding those in mitochondrial and apicoplast genomes. The genes were functionally annotated, and Gene Ontology (GO) terms are shown in supplementary table S2, Supplementary Material online.

#### Plasmodium *Gonderi pir* Gene Family

An example of this genome-improved annotation is the *Plasmodium interspersed repeat* (*pir*) family, which has a suspected role in virulence and chronic infection, particularly in nonfalciparum *Plasmodium* species infecting mammals (Harrison et al. 2020; Little et al. 2021). Many *pir* genes are species-specific, requiring high-quality genome assemblies to characterize them. Given the limited high-quality genome data for many *Plasmodium* species, the number of *pir* genes shared between closely related species is usually underestimated. Nevertheless, it is worth noting that it is not possible to find *Pir* orthologous genes across the genus (Harrison et al. 2020).

The *P. gonderi* v1 assembly shows 298 (of 914) annotated *pir* genes on chromosomes with a scattered distribution (supplementary fig. S2, Supplementary Material online). In contrast, the *P. gonderi* v2 assembly has 661 (out of 1160) *pir* genes on chromosomes distributed mainly in subtelomeric regions (supplementary fig. S2, Supplementary Material online), as observed in other *Plasmodium* assemblies with high coverage. The remaining *pir* genes from *P. gonderi* v2 are on 9 contigs not assigned to specific chromosomes. A similar pattern was previously reported in the *P. cynomolgi* genome (Pasini et al. 2017; supplementary fig. S3, Supplementary Material online).

**Fig. 1.**
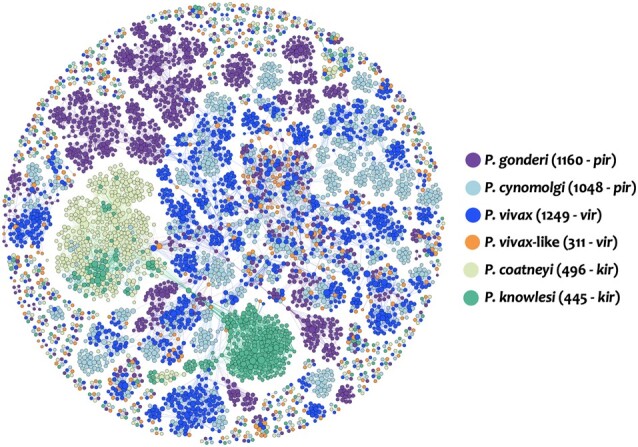
Network plotting the pir gene proteins similarity between *P. gonderi* (1160 pir), *P. cynomolgi* M (1048 pir), *P. coatneyi* Hackeri (496 kir), *P. knowlesi* H (445 kir), *P. vivax*-like Pvl01 (311 vir), and *P. vivax* P01 (1249 vir). The size of each node is determined by the number of edges per node (or gene). The more edges a node has, the larger its size.

Figure 1 shows clusters obtained using reciprocal Basic Local Alignment Search Tool (BLAST) of *pir* genes from *P. gonderi* v2 and those found in other related *Plasmodium* species expected to be in the same clade; several *pir* paralogs form clusters within rather than between species. This pattern indicates a fast rate of evolution in terms of contraction/expansion of the gene family, as reported elsewhere (Pasini et al. 2017). Clusters with 1 or more *pir* genes from more than one species suggest they may be conserved (Neafsey et al. 2012; Little et al. 2021), but those are usually considered very few. However, approximately 10% of *P. gonderi pir* genes reported in this new genome (114 out of 1160) form small clusters of 5 to 8 pirs, with genes from 5 or all 6 species included in this comparison (Fig. 1). Pirs from *P. vivax-*like are usually missing from those clusters where *P. gonderi* and the other species are represented, perhaps due to genome quality issues as will be shown later. Notably, most putatively conserved *pir* genes (105 out of 114 in *P. gonderi*) are mapped in chromosome 1. A similar pattern is observed in *P. vivax* and related species with assemblies carried out independently from this study (e.g. 115 out of 120 conserved *pir* genes in *P. vivax* chromosome 1) (Auburn et al. 2016; Chien et al. 2016; Pasini et al. 2017; Lapp et al. 2018). Whether the conservation regarding similarity and chromosome allocation of these *pir* genes within this clade reflects their functional importance needs to be determined (Little et al. 2021). Overall, improving the genome assembly (supplementary table S1, Supplementary Material online) allowed us to describe this gene family better as an example of how this resource will facilitate comparative genomic studies.

#### GC Content among *Plasmodium* Genomes

**Fig. 2.**
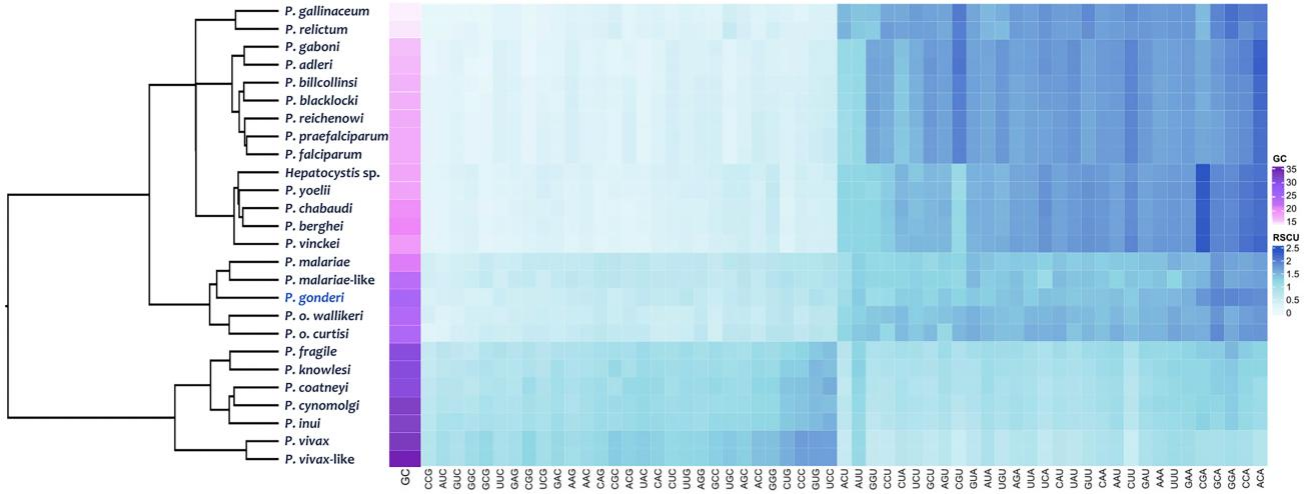
Hierarchical cluster by average and heat map of RSCU values of each codon in the SC-OGs of haemosporidian genomes. Each square in the heat map represents each codon RSCU value (in rows) within the SC-OG of each genome (in columns). Colors indicate the magnitude of RSCU values. A color bar indicates the species genome GC content.

There are 26 *Plasmodium* species with genomic assemblies of various qualities (supplementary table S1, Supplementary Material online). Two sets of loci were considered to investigate the GC content and the Relative Synonymous Codon Usage (RSCU). One is limited to 1,961 single-copy orthologous genes (SC-OGs) found in all species, and the other includes all CDSs available for each species regardless of the quality of the genome data (supplementary table S3, Supplementary Material online). The GC content and cluster analysis with their RSCU values on the SC-OGs are depicted in Fig. 2. All *Plasmodium* sequences have low GC content. However, there were marked differences among the species. For example, GC was twice as high in *P. vivax* (45.76% for CDS and 34.45% in SC-OGs) as in *P. gallinaceum* (22.54% for CDS and 15.20% in SC-OGs). The same differences were observed in total genomic GC content and noncoding regions (Fig. 2, supplementary fig. S4 and table S1, Supplementary Material online): a lower GC content in the subgenus *Laverania* and a distinct relatively high GC pattern in *P. vivax* and species found in Southeast Asia (subgenus *Plasmodium*), setting them apart from other nonfalciparum primate parasites from Africa, including *P. gonderi*. Such a pattern suggests some form of mutational bias. However, more data is needed to test specific hypotheses, such as GC content association with recombination rates per time unit across the genus *Plasmodium* (Romiguier and Roux 2017).

Although not a phylogenetic analysis, the clustering of RSCU per codon using SC-OGs (Fig. 2) or all CDS (supplementary fig. S4, Supplementary Material online) showed patterns consistent with a phylogenetic signal. The previously reported monophyletic group that includes *P. falciparum*, which will henceforth be called *Laverania*, exhibited a similar low GC content and codon bias. *Laverania* has similar GC content and codon bias compared to the 2 avian parasites (*Plasmodium relictum* and *Plasmodium gallinaceum*) (Böhme et al. 2018) and the rodent parasites (supplementary table S4, Supplementary Material online). *Hepatocystis,* a genus of haemosporidian parasites with distinct life cycles (Garnham 1966), was placed within this group in the RSCU cluster analysis, presenting similarly low GC content.

The clustering of RSCU per codon also recovered 2 groups of primate parasites that are not related to *P. falciparum*. One cluster shows *P. gonderi* with *P. malariae* and *P. ovale s.l*. However, clearly outside the cluster that includes species from Southeast Asia, *P. vivax*, and *P. vivax-*like (Fig. 2). *Plasmodium vivax* (45.76%) and *P. vivax-*like were more GC-rich than other species from Southeast Asia (38.89% to 42.95%, supplementary table S1, Supplementary Material online). They were also biased toward codons CUG, CCC, GUG, and UCC, which correspond to the amino acids Leu, Pro, Val, and Ser (supplementary table S4, Supplementary Material online). Such patterns may translate into the rate heterogeneity reported in other studies affecting phylogenetic inferences with a limited number of genes and sites per gene (Galen et al. 2018). The same clustering emerged when analyzing the second set of loci that included all the CDSs available for each species (supplementary fig. S4, Supplementary Material online).

It is worth noting that the SC-OGs (found in all species) have lower GC content than all CDSs (supplementary table S1, Supplementary Material online). Such a difference might indicate that genes conserved across *Plasmodium* may have higher codon bias due to selected efficiency in their expression because they are essential (Zhou et al. 2016; Oberstaller et al. 2021). However, the low quality of some genomes and the lack of functional genomic data in many parasites do not allow comprehensive analyses.

*Plasmodium gonderi* clustered with *P. malariae* when analyzing the frequency of amino acids in the proteome and SC-OGs (supplementary fig. S5 and table S5, Supplementary Material online). In the same analysis, *P. ovale s.l.* was placed in the same cluster as the rodent malaria parasites and *Hepatocystis* sp., parasites found in nonhuman primates and bats. Although cluster analyses are not phylogenies, these patterns on amino acids may explain the findings of previous studies, where *P. ovale* seems to share a common ancestor with rodent parasites (Sharp et al. 2022), mainly when working with proteins instead of DNA.

In summary, cluster analyses of codon usage and proteome place *P. gonderi* with other primate-infecting, nonfalciparum-related *Plasmodium* species that originated in Africa but set it apart from Southeast Asian parasites. Discrepancies between the clustering in the proteome and the codon usage analyses may indicate underlying processes that affect phylogenetic inferences if those differences are not considered a priori.

#### Synteny among *Plasmodium* Genomes

**Fig. 3.**
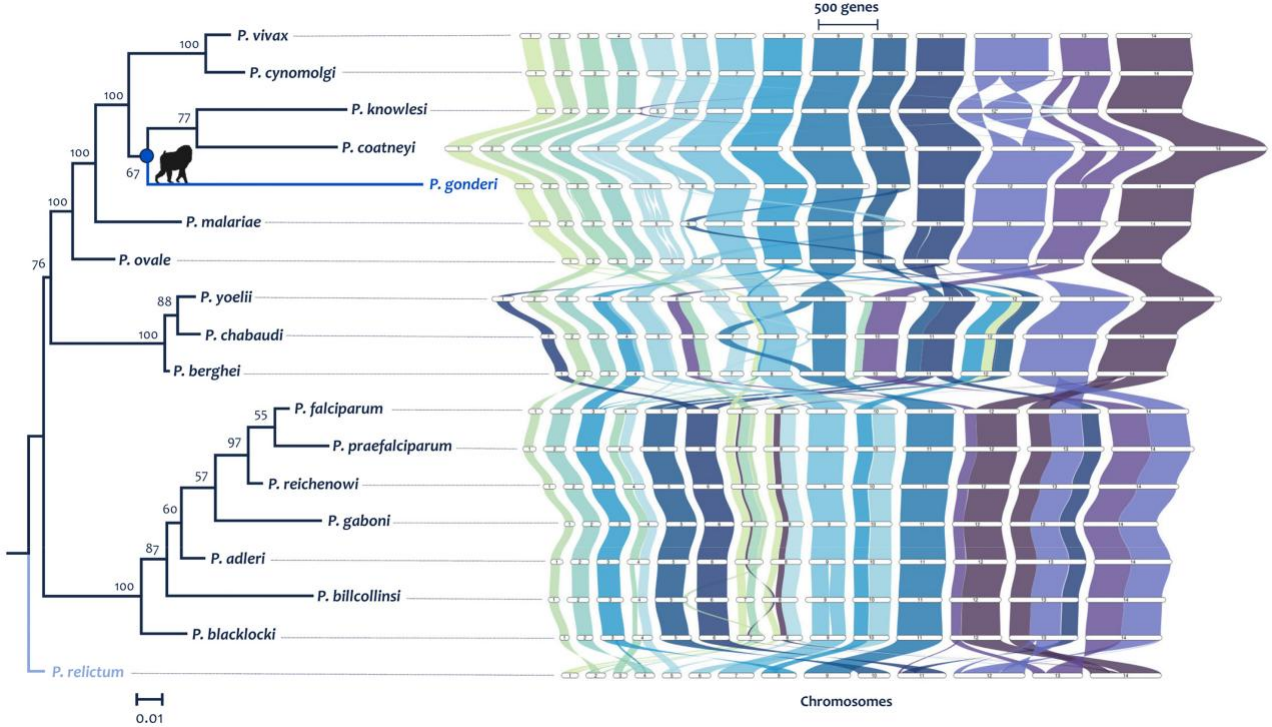
Phylogenomic reconstruction based on synteny and graphical representation of the syntenic blocks across the plasmodium genus. Phylogenetic reconstruction based on synteny was inferred by maximum likelihood methods implemented in IQ-tree software (100 bootstrap replicates) from the synteny matrix obtained following the protocol of Zhao et al. (2021). The synthetic blocks were graphed with the R package GeneSpace (Lovell et al. 2022). The corrected assembly of *P. coatneyi* Hackeri is used here.

Synteny among the *Plasmodium* genomes was studied on 18 species out of 26 genomes since they have chromosome-length or less fragmented assemblies (supplementary table S1, Supplementary Material online). Visual inspection indicated major syntenic blocks shared between *Plasmodium* species (Fig. 3). However, rearrangements that separated species clades were evident as the rodent malaria, the *Laverania* clade containing *P. falciparum*, and a primate parasite clade with all the other human malaria showing apparent differences in synteny. There were a few translocations, insertions, and deletions of large syntenic blocks between *P. gonderi* and other *Plasmodium* species found in primates from Southeast Asia and *P. vivax*. Further exploration requires determining whether there are still quality issues in some genome assemblies that could lead to the generation of putative chromosome rearrangements. E.g., the *P. coatneyi* genome, as available in databases, showed genome rearrangements in chromosomes 2, 13, and 14 compared with the other species found in macaques (supplementary fig. S6, Supplementary Material online). Such rearrangements are likely artifacts. Indeed, they disappeared when the *P. coatneyi* genome was assembled de novo using the same methodologies reported here for *P. gonderi* (Fig. 3 and supplementary fig. S6, Supplementary Material online). Putative assembly artifacts also could be observed among the rodent malaria parasites and the macaque parasite *P. knowlesi* (inversions in chromosome 12).

Regardless of assembly concerns, a synthetic-based phylogenomic reconstruction (Fig. 3 and supplementary fig. S6, Supplementary Material online) supported previous phylogenetic inferences (Escalante and Ayala 1994; Escalante et al. 1995; Nishimoto et al. 2008; Pacheco et al. 2018; Escalante et al. 2022; Pacheco et al. 2022). The avian parasite *Plasmodium relictum* was used as the outgroup based on findings from single locus and multilocus phylogenies estimated on several Haemosporida species (Martinsen et al. 2008; Galen et al. 2018; Pacheco et al. 2018, 2022). *Plasmodium gonderi* shares a common ancestor with Southeast Asian parasites, and *P. ovale* and *P. malariae* form a clade with other primate parasites. However, the synteny phylogeny presented here lacks critical species such as *P. vivax-*like. Although a genome is available, *P. vivax-*like produced peculiar patterns likely due to assembly problems (see supplementary fig. S7, Supplementary Material online, which includes both assemblies of the 2 available *P. vivax*-like lineages).

*Plasmodium relictum,* the only avian parasite with a high-quality genome (Böhme et al. 2018), showed rearrangements not observed in parasites found in primates and rodents. However, many other avian genomes are needed to identify patterns since the available evidence indicates that these parasites are more diverse than those found in mammals (Valkiūnas, 2005; Valkiūnas and Iezhova 2018).

A previous study (Zhang et al. 2018b) hypothesized that syntenic regions have more critical genes based on the growth of *P. falciparum* asexual stage in vitro. In contrast, nonsyntenic blocks were enriched with “dispensable” genes, including genes encoding proteins considered virulence factors. The syntenic regions were relatively few inferred in a comparative genomic study with limited well-annotated *Plasmodium* genomes (DeBarry and Kissinger 2011). This hypothesis was further tested using a parsimony-based algorithm implemented in the software AGORA (Muffato et al. 2023) that estimated the content and gene order in the ancestor from the 18 complete genomes of *Plasmodium* species, defining syntenic block across all species, including *P. gonderi*. A GO enrichment analysis (using a Fisher's exact test and Bonferroni correction) identified the functional categories of each gene present in both the syntenic blocks and the breakpoints based on the classification from Zhang et al. (2018b) as essential genes (supplementary table S6, Supplementary Material online). The pattern was explored on the common ancestors of *Laverania,* as was *P. falciparum* where the functional analyses were performed, then all mammalian *Plasmodium,* and finally all *Plasmodium* species were included in the analysis. In all these 3 comparisons, syntenic blocks harbor more putatively essential genes, as defined by Zhang et al. (2018b), than expected by chance (supplementary fig. S8, Supplementary Material online).

However, a bias exists in focusing on *P. falciparum* data from an in vitro culture. Alternatively, it can be posited that orthologous genes among all *Plasmodium* species are putatively essential (Kooij et al. 2005; Oberstaller et al. 2021). The putative *Plasmodium “*core genome” comprised 3,408 SC-Ogs in these 18 *Plasmodium* reference genomes. Of those, >98.9% of the genes are located in the syntenic blocks depending on the clade (supplementary table S7 and fig. S9, Supplementary Material online). In contrast, several genes involved in the vertebrate host-parasite relationship were in the more plastic part of the genome or breakpoints outside the syntenic blocks (supplementary table S8 and fig. S9, Supplementary Material online). This pattern may reflect the observed biological diversity of *Plasmodium* in mechanisms to invade the vertebrate host cell and evade the host immune response, as previously suggested with a limited dataset (Kooij et al. 2005) (supplementary table S8, Supplementary Material online).

**Fig. 4.**
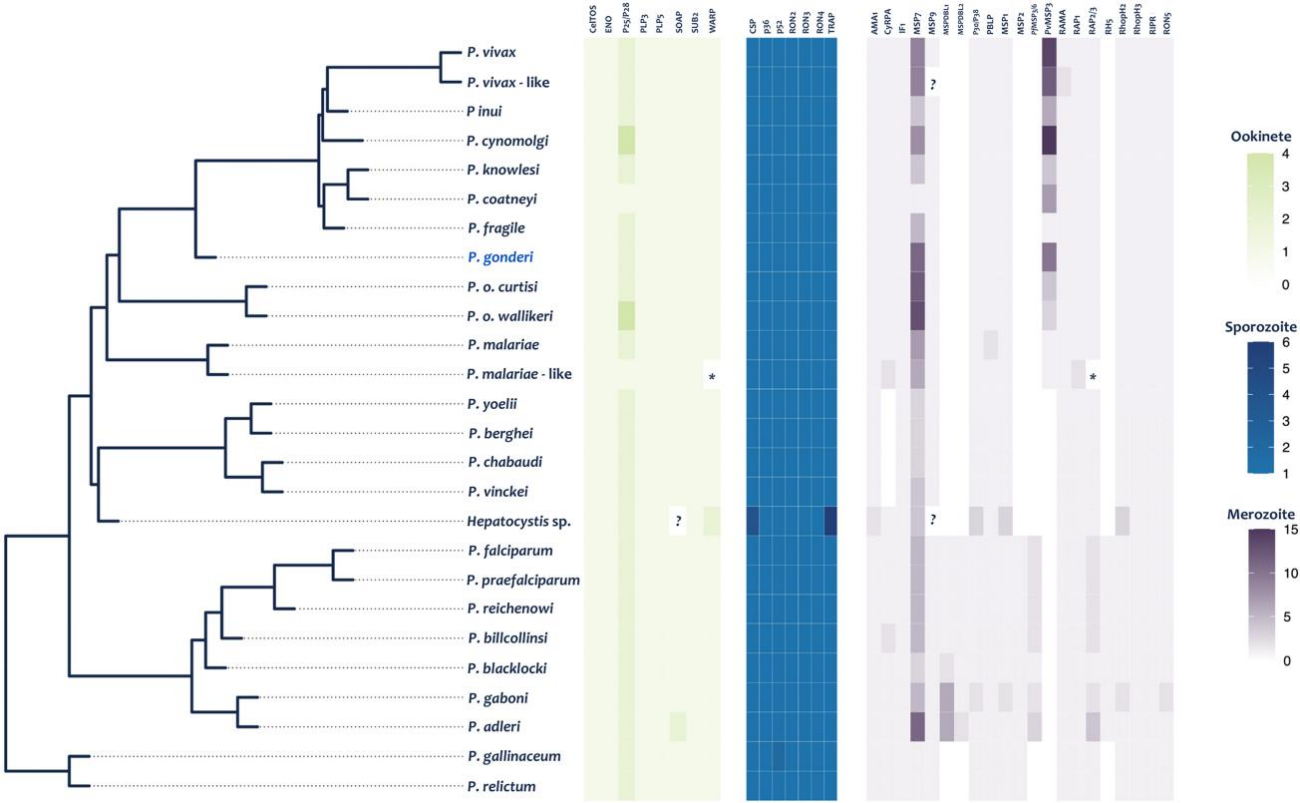
Mapping of genes involved in the parasitic stages of cell invasion. The phylogeny used corresponds to the species tree previously reported (Escalante et al. 2022). Each cell in the parasitic stages heatmaps corresponds to the number of gene copies for each gene per species. (*) Genes that are present but incomplete and (?) missing genes in the genome. The genes functions are described in Kaur et al. (2022), Graumans et al. (2020), and Wright and Rayner (2014) for the ookinete, sporozoite, and merozoite, respectively.

Although identifying conserved genes across *Plasmodium* seems a more robust approximation to define an essential gene than just data from one species in culture, it still overlooks *Plasmodium* species’ divergent life histories resulting in clade-specific genes. For example, Fig. 4 shows a small sample of functionally important genes expressed in different life cycle stages. Those genes were chosen because they are involved in processes considered fitness components, like the invasion of the red blood cell or passage of the oocyst (vector stage) through the *Anopheles* midgut in the different *Plasmodium* species (see Graumans et al. (2020), Kaur et al. (2022), and Wright and Rayner (2014) for genes expressed in the ookinete while in the mosquito vector, the sporozoite or infectious stage, and the blood stage or merozoite, respectively). As can be observed, some genes, particularly those involved in the invasion of red blood cells, do not have orthologs across the genus, such as the gene encoding RH5, essential in the infection of the red blood cell by *P. falciparum* (Wright and Rayner 2014; Plenderleith et al. 2018). Thus, the observed pattern of conserved genes in syntenic blocks must be interpreted considering that it does not account for clade-specific essential genes.

Overall, syntenic blocks provide phylogenetic information that places *P. gonderi* in a clade with *P. vivax* and other nonhuman primate parasites from Southeast Asia. Syntenic blocks are enriched with conserved genes detected across *Plasmodium* species. In contrast, genes associated with the host-parasite interaction, such as gene families involved in virulence, are outside such blocks (Kooij et al. 2005). This observation suggests that rearrangements in nonsyntenic regions are critical in the vertebrate host-parasite relationships.

#### Phylogenomic Reconstructions

A total of 1,961 single-copy gene ortho groups (SC-Ogs) found across all 26 species were included in this study (supplementary table S3, Supplementary Material online). Given that 3,408 SC-Ogs were recovered from 18 complete genomes, it is clear that some draft genomes were not only fragmented but incomplete (e.g. *Hepatocistys* sp., *P. vivax*-like, and *P. fragile*). As IQ-tree (Nguyen et al. 2015; Minh et al. 2020) estimated, 3 substitution models accounted for 91.82% of the total gene alignments (56.85% general time reversible (GTR), 29.57% TIM3, and 5.4% TVM; supplementary table S3, Supplementary Material online).

**Fig. 5.**
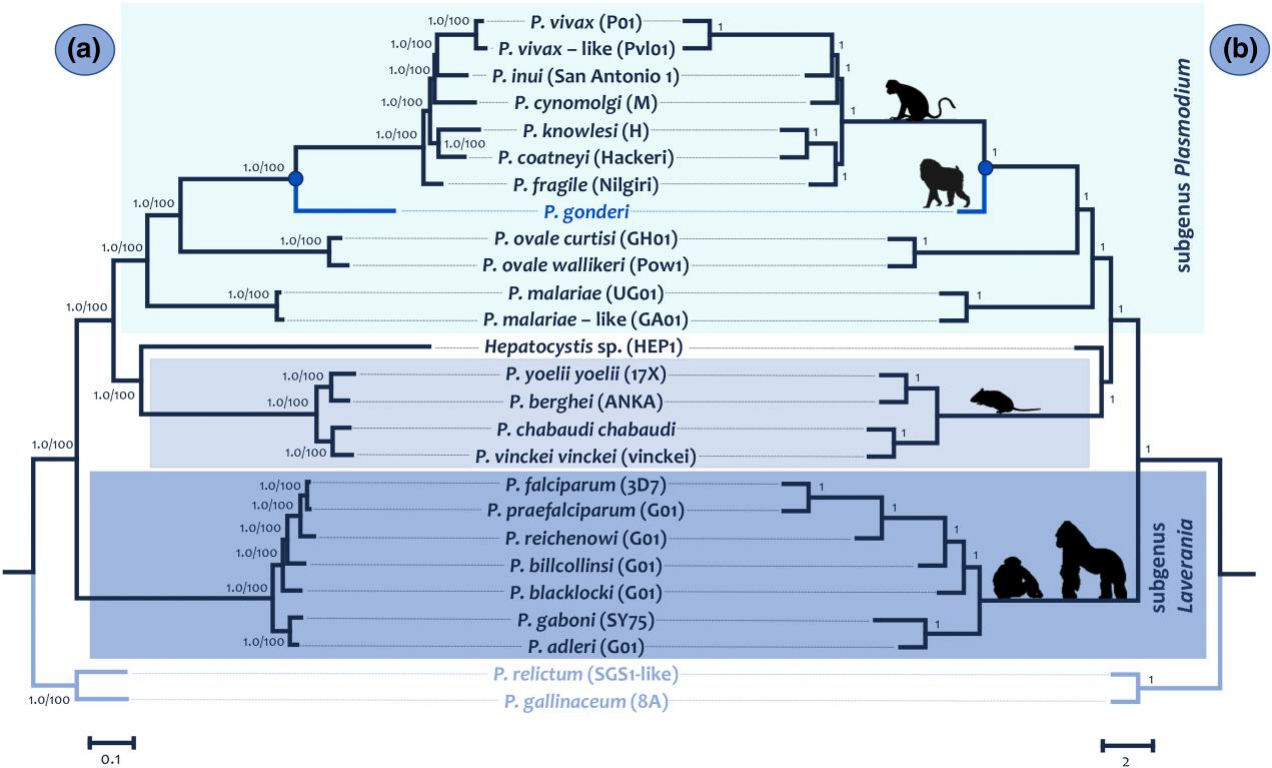
Species trees: a) phylogeny based on the concatenated alignment of the 1,961 SC-OGs using the Bayesian methods implemented in MrBayes v3.2.6 with the default priors (Ronquist and Huelsenbeck 2003) and the ML method in IQ-Tree (Minh et al. 2020). All nodes show posterior probabilities and bootstrap values as a percentage obtained for 1,000 pseudoreplicates. b) Phylogeny based on the multispecies coalescent model implemented in ASTRAL-III (Zhang et al. 2018a, 2018b) from the 1961 SC-GO trees. All nodes show values of posterior probabilities.

*Plasmodium* species trees were first inferred from a concatenated alignment (2.4 Mb without gaps) using Bayesian and Maximum Likelihood (ML) methods (Fig. 5a) with the GTR + I + G4 substitution model, as determined using IQ-tree. The species tree was also inferred using the multispecies coalescent model (Fig. 5b) implemented in Astral III using SC-OG trees estimated with IQ-tree under the best-fitted model (supplementary table S3, Supplementary Material online). The phylogenies obtained were almost identical and well-supported (Fig. 5); the exception was the fragile position of *Plasmodium*. The results were reproduced using codon (not included) instead of nucleotide models yielding the phylogeny of the concatenated alignment. It is worth noting that using normalized Robinson–Foulds distances (nRF), a paired comparison was made between each gene tree topology calculated by IQ-tree and the species tree (supplementary table S3, Supplementary Material online). The mean of these distances was 13.25 (s^2^ = 17.3), indicating that, on average, there are 8 changes between a gene tree and the estimated species tree.

Non-Laverania *Plasmodium* species found in primates, including *P. gonderi*, belonged to a monophyletic group in this analysis. This clade shared a common ancestor with rodent malarias and *Hepatocystis* sp. (Fig. 5). The relative position of *Hepatocystis* sp. is consistent with previous studies (Aunin et al. 2020; Escalante et al. 2022). However, it is worth reiterating that several species of *Plasmodium* in nonhuman primates have no genomic data available including all parasites from gibbons, orangutans, and lemurs (Coatney et al. 1971; Peters 1976; Pacheco et al. 2022). Other *Plasmodium* species found in bats and adding more species of *Hepatocystis* sp. may also change this result, particularly the rodent parasites clade (Schaer et al. 2013).

The phylogenomic analyses (Fig. 5) placed the common ancestor of *P. vivax* and *P. vivax-*like as part of the parasite radiation of nonhuman primates from Asia, supporting earlier studies that only included *P. vivax* (Escalante et al. 2005; Mu et al. 2005; Pacheco et al. 2012; Prugnolle et al. 2013; Muehlenbein et al. 2015; Pacheco et al. 2018). Although information from other parasite species found in macaques or gibbons could change this phylogeny, the tree topology is consistent with other studies with more *Plasmodium* species but that used single gene or multilocus approaches (Escalante et al. 2005; Nishimoto et al. 2008; Mitsui et al. 2010; Pacheco et al. 2018; Pacheco et al. 2022; Pacheco and Escalante 2023). It is consistent with the phylogenetic hypothesis emerging from studying syntenic blocks. Still, it differs from other studies where the *vivax* lineage appears as a sister clade to the other macaque parasites (Sharp et al. 2020; Arisue et al. 2022). The discrepancy may be in the methods used to reconstruct the phylogeny and the data considered. Here, DNA sequences were chosen a priori as they are more informative (Hall 2005; Quek and Huang 2019; Young and Gillung 2020).

*Plasmodium cynomolgi* has been considered the species from Southeast Asia that shared the most recent common ancestor with the lineage leading to *P. vivax*, an inference based on biological traits (Collins and Contacos 1980; Tachibana et al. 2012; Pasini et al. 2017; Galinski 2022). Finding *Plasmodium inui* sharing a more recent common ancestor with the *P. vivax* lineage contradicts that hypothesis. A better species sampling may be required (Muehlenbein et al. 2015). The lack of genomic data on parasites from gibbons or orangutans may have affected this phylogeny (Pacheco et al. 2012; Muehlenbein et al. 2015; Pacheco et al. 2018).

The inclusion of *P. gonderi* in the phylogenies inferred here showed that the common ancestor between *P. vivax* and *P. vivax*-like may have arisen during the radiation of the species currently found in nonhuman primates from Southeast Asia. These results coincide with population genomic and single gene studies indicating that *P. vivax* originated in Asia (Cornejo and Escalante 2006; Prugnolle et al. 2013; Daron et al. 2021). Thus, based on the phylogenies presented here, the species called *P. vivax-*like is likely an early introduction of the *P. vivax* lineage from Asia into Africa.

#### Time-Tree Analyses

Estimating time trees with several hundreds of genes is impractical (Battistuzzi et al. 2018; Tamura et al. 2018). Identifying genes with similar GC content (Romiguier and Roux 2017; Quek and Huang 2019; Kapli et al. 2020) among *Plasmodium* species was impossible (see supplementary tables S3 and S9, Supplementary Material online). Thus, 110 genes were chosen using the smaller nRF distances between gene and species trees. This subset yields a similar phylogeny obtained with the 1,961 SCGOs (Fig. 5).

Informative calibrations are critical to obtaining reliable absolute timescales in relaxed molecular clock methods (dos Reis et al. 2016). A problem when timing *Plasmodium* in primates, in addition to differences in GC content among clades, is that no parasite fossils can provide primary calibrations (Pacheco and Escalante 2023). Thus, assumptions based on the association between parasite clades and hosts’ events must be made, making such constraints a form of secondary calibrations. The use of secondary calibrations is controversial but, in this case, unavoidable (Powell et al. 2020). Potential problems can be mitigated by comparing different calibration strategies (Pacheco et al. 2018, 2022). Overall, calibrations using the host should be as inclusive as possible to avoid unrealistic precision (Graur and Martin 2004; Pacheco et al. 2018).

Relaxed clock methods differ in how evolutionary rate variation among lineages is modeled as autocorrelated or independent (uncorrelated) (dos Reis et al. 2016; Battistuzzi et al. 2018). Such differences have proven to affect time estimates in *Plasmodium* (Pacheco et al. 2018). Here, the effect of rate variation models was explored using MCMCTree and 3 different calibration scenarios (with one, two, and 3 calibration constraints). Then, MCMCTree estimates were compared against the results from a non-Bayesian dating method that estimates relative divergence times, RelTime (Tamura et al. 2012). Supplementary table S10, Supplementary Material online shows all the estimated node ages in MCMCTree and their credible intervals (CrI) under the different calibration scenarios. All calibration scenarios were compared using autocorrelated and uncorrelated models (supplementary fig. S10, Supplementary Material online indicates the node numbering in supplementary table S10, Supplementary Material online).

The first scenario used a single calibration prior that considers the divergence of African parasites found in *Mandrillus* spp. and *Cercocebus* spp. (*P. gonderi*) from those *Plasmodium* species found in Southeast Asian macaques (Mu et al. 2005; Pacheco et al. 2011). The second scenario (supplementary table S10, Supplementary Material online) also added a calibration at the origin of the monophyletic group that includes *P. malariae* (a parasite found in humans). In particular, a minimum of 23.5 MYA, which corresponds to the human/*Macaca* split (Benton and Donoghue 2007), with a maximum of 65 MYA, this calibration allows this clade to be as old as the origin of primates (Perelman et al. 2011; Ksepka et al. 2015; Álvarez-Carretero et al. 2022). Both scenarios yield consistent time estimates whether the independent (*R*^2^ = 1.0) or autocorrelated models were used (*R*^2^ = 1.0) (supplementary figs. S11 and S12, Supplementary Material online). Furthermore, RelTime and MCMCtree yield consistent time estimates (*R*^2^ = 0.99 when using both scenarios with the 2 Bayesian models). Thus, the scenario using 2 calibrations on Bayesian models is discussed in detail (Table 1, supplementary fig. S12, Supplementary Material online).

**Table 1:** Molecular time estimates for major events during the evolution of *Plasmodium* in primates contrasting autocorrelated and independent (uncorrelated) rate models

	Scenarios							
	Correlated		Independent		Correlated		Independent	
Calibrations (Ma)	Scenario I: 1: 6–14.2 2: 24.44–34.0				Scenario II 1: 6–14.2 2: 24.44–34.0 3: 6.5-15.2			
Divergence	Node age	95% CrI	Node age	95% CrI	Node age	95% CrI	Node age	95% CrI
Origin of *Plasmodium* spp./*Plasmodium vivax* (human and Asian NHP)	3.44	3.30 to 1.63	5.77	7.92 to 3.83	4.11	5.77 to 2.55	6.0	7.38 to 4.56
Divergence between *P. vivax* and *P. vivax*-like	0.29	0.50 to 0.11	0.76	1.33 to 0.29	0.34	0.56 to 0.17	0.80	1.35 to 0.33
Origin of *Plasmodium gonderi* (African NHP)	11.14	14.42 to 7.37	10.88	14.28 to 7.38	13.31	14.82 to 11.09	13.29	14.81 to 11.13
Origin of *Plasmodium ovale* (human)	23.95	29.39 to 19.06	23.75	29.80 to 18.29	24.63	29.29 to 20.60	25.19	30.28 to 20.20
*Plasmodium ovale* subspecies divergence	2.88	5.83 to 0.62	2.37	4.26 to 0.87	2.82	5.42 to 0.76	2.52	4.33 to 1.04
Origin of *Plasmodium malariae* (human and NHP)	28.71	33.69 to 24.30	29.07	33.79 to 24.38	28.95	33.79 to 24.42	30.39	34.28 to 25.51
*Plasmodium malariae—malariae -*like divergence	1.00	2.42 to 0.11	0.38	0.69 to 0.13	0.92	2.17 to 0.12	0.41	0.71 to 0.16
Origin of *Hepatocystis* spp. (NHP)	28.08	34.34 to 22.61	28.44	35.89 to 20.95	28.02	34.21 to 22.57	29.75	37.18 to 21.95
Origin of *Plasmodium* spp. (rodents)	6.46	11.06 to 2.24	6.07	9.21 to 3.44	5.23	8.30 to 2.13	5.81	7.87 to 3.70
Origin of *Laverania* genus	9.75	15.02 to 4.63	5.27	7.93 to 3.0	6.98	8.84 to 5.56	6.68	7.96 to 5.55
Origin of *Plasmodium* genus + *Hepatocystis* spp. (primates and rodents)	40.75	50.19 to 31.83	40.4	49.72 to 31.79	42.24	55.75 to 30.52	43.03	52.57 to 33.28

The node ages and their credible intervals are provided for the 2 and 3 calibration scenarios.

Time estimates derived from nuclear genes did not deviate from those based on organelle genomes for several nodes, even when there are differences in GC content among clades in the nuclear genes. The estimates for the origin of *Plasmodium* found in primates and rodents were similar between the autocorrelated and independent models, 40.75 MYA (CrI = 50.20 to 31.83) and 40.40 MYA (CrI = 49.72 to 31.79), respectively. These time estimates were consistent with studies using the parasite mtDNA (Pacheco et al. 2018; Pacheco et al. 2020) and combining the mtDNA and loci from the apicoplast (≅6 kb) (Pacheco et al. 2022), a plastid-like organelle found in Haemosporida, which has a circular genome with similar AT content to the mtDNA genome (Wilson et al. 1996).

The split of the lineage leading to *P. ovale* s.l. species is dated at 23.95 MYA (CrI = 29.39 to 19.06) for autocorrelated and 23.75 MYA (CrI = 29.80 to 18.29) for uncorrelated (Table 1, supplementary fig. S12, Supplementary Material online) rate models. These estimates are consistent with target gene approaches on organelles using different assumptions (Hayakawa et al. 2008; Sutherland et al. 2010; Pacheco et al. 2018, Pacheco et al. 2022) and suggest that the lineage leading to *P. ovale* s.l. diverged from the other *Plasmodium* included in this analysis early during the origin of Catharrini primates (Benton and Donoghue 2007). The mean posterior divergence time between the 2 *P. ovale* cryptic species was estimated to be between 2.52 and 2.88 MYA, depending on the substitution rate model and calibration scenario (Table 1, supplementary fig. S12a, Supplementary Material online). These coincide with estimates based on mitochondrial loci using *Laverania* species as calibration (Sutherland et al. 2010) and are consistent with the proposed timeframes for the origin of the genus *Homo* (Püschel et al. 2021). These results contrast with a genomic study that estimated the split of the *P. ovale* s.l. species at 20.3 MYA (Rutledge et al. 2017). Although the methods and assumptions could partially explain the differences, the estimate seems unrealistic, considering the 2 cryptic species’ genomic divergence and biological similarities.

The mean posterior divergence time for the split of *P. vivax* and *P. vivax*-like is between 0.29 MYA (CrI = 0.50 to 0.11) in the autocorrelated model and 0.76 MYA (CrI = 1.33 to 0.29) in the independent model (Table 1). These estimates place this divergence at a time when environmental changes connected Asia and Africa, allowing hominids to migrate across (Roberts and Stewart 2018; Louys and Roberts 2020; Groucutt et al. 2021; Lee and Hudock 2021). Thus, the inferred divergence time between *P. vivax* and *P. vivax-*like, together with the placement of their common ancestor within the radiation of the *Plasmodium* species found in Southeast Asia, is consistent with a scenario where *P. vivax* originated in Asia as a human parasite as suggested by population genetics and genomic analyses on the extant populations of *P. vivax* (Cornejo and Escalante 2006; Benavente et al. 2021; Daron et al. 2021). Although an African origin (Loy et al. 2018) seems less parsimonious in light of the population genetics and phylogenetic data, it does not make it impossible. The *P. vivax-*like genomic data are still scarce, and data from *P. vivax* infections in Africa are still being studied by the scientific community. However, data indicating a more ancient *P. vivax* population in Africa is still not available so the African scenario seems less parsimonious just when considering the available data.

An Asian origin hyphotesis for *P. vivax* is usually criticized because it cannot explain the high prevalence of Duffy-negative in African human populations. Duffy-negative individuals are protected against the blood stage of *P. vivax*, and it is considered to have been selected by the parasite (revised in McManus et al. 2017) and explained the apparent absence of *P. vivax* in sub-Saharan Africa. However, unlike polymorphism protecting against *P. falciparum* malaria (Malaria Genomic Epidemiology Network 2019), the expected patterns consistent with the strong selection of these Duffy-negative alleles remain elusive, and the origins of all Duffy-negative haplotypes seem consistent with the timeframes estimated in this study (McManus et al. 2017). The situation is further complicated because *P. vivax* can be maintained in Duffy-negative African populations (Ménard et al. 2010; Gunalan et al. 2018). Although Duffy-negative polymorphisms in Africa are driven by evolutionary processes that likely correlate with what we observe in *P. vivax* today, establishing causation is not obvious (Gould and Lewontin 1979; Hughes and Verra 2010) so it cannot be used as evidence supporting a particular scenario for the origin of *P. vivax* as a human parasite.

The estimates for the origin of *Laverania*, the clade of *P. falciparum* and related species, differed between the autocorrelated (9.75 MYA, CrI = 15.02 to 4.63) and independent (5.27 MYA, CrI = 7.93 to 3.00) models. However, their CrI overlapped (Table 1, supplementary fig. S12, Supplementary Material online). These estimates coincided with the origins of their hosts (e.g. median 8.6 MYA, CrI = 8.3 to 9.3 MYA for the common ancestor of *Gorilla* and *Homo*, based on the TimeTree database accessed on August 2023, Kumar et al. 2022). Similar time estimates were also obtained when combining organelle loci from mtDNA and apicoplast, or studies on nuclear loci (Hughes and Verra 2010; Pacheco et al. 2022). These results differ from studies proposing that the *Laverania* clade originated less than 1 MYA ago (Otto et al. 2018) when *Homo, Gorilla,* and *Pan* coexisted (Bobe and Reynolds 2022; Urciuoli and Alba 2023). It implies that the local ecology drove the clade speciation (Makanga et al. 2016; Liu et al. 2017). However, Otto et al. (2018) used different assumptions in a coalescent-based approach suitable for intra-species variation rather than species divergence. Under such a model, all *Laverania* seems to be treated as a single species. In contrast, this investigation using models to study species divergence supports a scenario where the African apes’ evolutionary histories may have played a role in the radiation of the *P. falciparum* clade. This study is consistent with the time proposed for the origin of the extant Laverania species but without formal molecular clock analyses being presented (Sharp et al. 2020). It is worth emphasizing that no calibration within *Laverania* was used to obtain the estimates. Such a calibration (e.g. *P. falciparum* and *P. reichenowi* divergence) is controversial (Sharp et al. 2020; Pacheco et al. 2022).

**Fig. 6.**
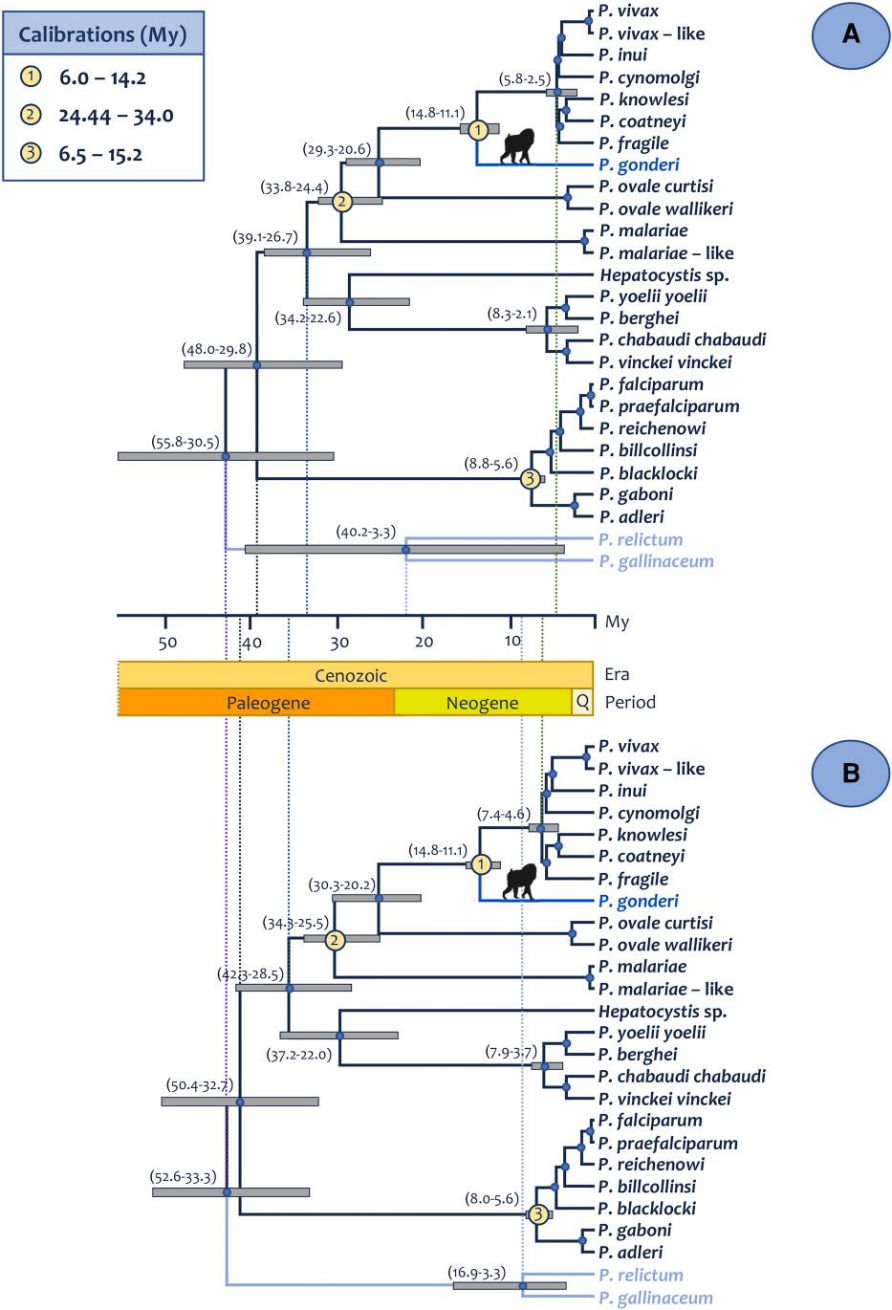
Time tree of the divergence of primate malarias using 110 genes. Divergence times were estimated using MCMCTree under the a) autocorrelated and b) independent rate models, using 3 calibration constraints that included a calibration for the origin of the Laverania clade. Calibrations priors were uniform, as explained in the text. Times are shown in MYA. 95% CrIs for the major clades are shown in parentheses next to the nodes.

Given the differences in time estimates between the Bayesian models, the method implemented in CorrTest (Tao et al. 2019), based on machine learning, was used to determine whether there was autocorrelation in substitution rates. CorrTest indicated that evolutionary rates are likely autocorrelated (CorrScore = 0.99845, *P* < 0.001). Considering that all the calibrations are in the “GC-rich” genomes, the effect of lacking calibrations within the *Laverania* subgenus, or *P. falciparum* clade, may have explained, at least in part, the difference between the 2 models. Thus, a third calibration was included at the origin of *Laverania* (Table 1 and Fig. 6) to explore its effect on the models. In particular, a minimum based on the fossil calibration between Chimps-*Homo* (6.5 MYA) and a maximum (15.2 MYA) using estimates for *Homo-Pongo* divergence (TimeTree database, Kumar et al. 2022). This calibration does not favor any particular event (uniform distribution). Such an interval includes recent estimates for the split between *Homo-Gorilla* and their crown groups (11.49 to 10 MYA; Püschel et al. 2021). It also allows the *Laverania* clade species to be as old as the origin of Hominidae, an unlikely scenario considering the extant *Plasmodium* species found in orangutans (Peters 1976; Pacheco et al. 2012). Adding this calibration yields an estimate of 6.98 MYA (CrI = 8.84 to 5.56) with the autocorrelated and 6.68 (CrI = 7.96 to 5.55) with the independent rate model. Thus, the uncertainty across the time tree decreases when a calibration constraint is added (even a broad one) in a clade that may be diverging from the others, *Laverania,* in this case, the one with lower GC content.

The medians of time estimates seem to favor the origin of *Laverania* during the *Homo­*-*Pan* divergence followed by host switches (fossils: 6.5 to 10 MYA sensu Benton and Donoghue 2007, http://fossilcalibrations.org last accessed on June 2022 and molecular estimates, median time = 6.4 MYA, CrI = 6.1 to 6.7 MYA; TimeTree accessed September 2022, Kumar et al. 2022). Such a scenario was proposed by others (Krief et al. 2010). The credible intervals, however, include the *Homo-Gorilla* divergence, particularly in the autocorrelated model that better fits the data. Also, estimates consistent with the *Homo-Gorilla* divergence emerged without calibration on *Laverania* under the autocorrelated model, which is a more conservative approach. Thus, the time frame presented here is consistent with a scenario where the extant host speciation played a role in the divergence of the *Laverania* parasites, masked partly by host switches during the evolutionary history of African apes.

### Overall Conclusion

The *P. gonderi* v.2 genome assembly presented here is a valuable resource for *Plasmodium* comparative genomics. *Plasmodium* species in primates and rodents have conserved syntenic blocks enriched by putatively essential genes. Notably, the genes outside such syntenic blocks seem critical to exploit their vertebrate hosts, as those with functional information are associated with virulence or host cell invasion. The observation is consistent in the diversity of strategies observed to circumvent the barriers involved in the infection of the vertebrate host.

Although contingent on the assumptions of this analysis, the emerging picture from this investigation is that the primate parasite host ranges and speciation, including the lineages leading to extant human parasites, may have been driven by their vertebrate hosts’ biogeographical and speciation processes. In particular, whereas the distribution and speciation of African apes may have driven the evolution of *P. falciparum* and related species, the origin of *P. vivax* is associated with the dynamics of primates in Southeast Asia and the connectivity of *Homo* populations between Asia and Africa. Several questions remain unanswered due to the absence of genome data from critical species found in macaques, gibbons, and lemurs. Nevertheless, the genome of *P. gonderi* has enriched our understanding of the evolution of *Plasmodium* in primates, particularly those infecting humans as their primary vertebrate host.

## Materials and Methods

### Sample and Ethical Considerations

*Plasmodium gonderi* was maintained in the Centers for Disease Control and Prevention by W.E. Collins. Strain Garnham is the only strain available (Collins and Contacos 1980). It can be found in the BEI Resources, NIAID, NIH, under the label: *P. gonderi*, Strain Garnham, MRA-447, and W. E. Collins contributed it. The DNA was extracted from a Centers for Disease Control and Prevention sample using QIAamp DNA Blood Mini Kit (QIAGEN, Germany), following protocols used in previous studies (Tachibana et al. 2012).

### Sequencing, Raw Data, Preprocessing, Assembly, and Annotation of *Plasmodium gonderi* Genome

The raw sequence data from the *P. gonderi* Sequence Read Archive (SRA) accession number SAMD00076127 (Honma et al. 2017) was combined with 3 paired-end next-generation sequencing libraries from the same strain; 2 with 100 bp (Illumina 2000) and 1 with 150 bp (Illumina 2500) read length (BioProject GenBank accession no. PRJNA928704). Low-quality read ends, and adapter sequences were removed using Trimmomatic software (Bolger et al. 2014). Trimmed reads were mapped to the *Macaca fuscata* genome (accession number GCA_003118495.1) using Bowtie2 with the default parameters (Langmead and Salzberg 2012) to eliminate reads that were likely from the host genome. Only unmapped reads against the host genome were used to run the assembly; ∼183 million reads were added from the 3 Illumina libraries produced in this study.

The genomic assembly (Genome Whole Sequence GenBank accession no. JARBEB000000000.1) was carried out by a de novo hybrid assembly strategy implemented in MaSuRCA software (Zimin et al. 2013). MaSuRCA was used with the following parameters: graph_kmer_size = 77, jf_size = 50000000, soap_assembly = 0, cgwerrorrate = 0.15, close_gaps = 1. This approach leads to fully assembled chromosomes (supplementary fig. S1, Supplementary Material online; Graphical representation was made using Circos software (Krzywinski et al. 2009)) and fully reproduced the synteny with *P. cynomolgi* without using any reference. The quality of the *P. gonderi* version 2 (*P. gonderi* v.2) genome assembly was evaluated and compared with 26 other haemosporidian genomes (including the draft assembly of *P. gonderi*, Honma et al. 2017) with Quast software (Gurevich et al. 2013; supplementary table S1, Supplementary Material online).

Structural annotation of genes was made using the Augustus gene prediction software (Stanke et al. 2006). Four genome annotations were used and compared to train Augustus: *P. cynomolgi* M (Pasini et al. 2017), *P. malariae* UG01 (Rutledge et al. 2017), *P. vivax* P01 (Auburn et al. 2016), and *P. knowlesi* H (Pain et al. 2008). However, training with *P. cynomolgi* showed the best results due to the number and length of genes. Manual corrections were made using Artemis software (Carver et al. 2005) comparing with closely related *Plasmodium* orthologous gene sequences. Functional and GO annotations were performed using the stand-alone software PANNZER2 (Protein ANNotation with Z-scoRE; Törönen et al. 2018; supplementary table S2, Supplementary Material online).

### Annotation of *Pir* Genes in Subtelomeric Regions

*Plasmodium*-interspersed repeat (*pir*) genes mapped on the chromosomes of the 2 *P. gonderi* genome assemblies were made employing the R package karyoploteR (supplementary fig. S2, Supplementary Material online; Gel and Serra 2017). Whereas there are multiple gene families, only *pir* gene family was included since it is informative about the genome quality. The following 6 genomes were used as they are part of the clase that includes *P. gonderi*: *P. coatneyi* Hackeri (Chien et al. 2016), *P. cynomolgi* M (Pasini et al. 2017), *P. gonderi* v2 (this study), *P. knowlesi* H (Pain et al. 2008), *P. vivax* P01 (Auburn et al. 2016), and *P. vivax-like* Pvl01 (Gilabert et al. 2018) (supplementary table S11, Supplementary Material online). The *Plasmodium* interspaced repeat (pir) protein sequences were extracted from the proteomes. Then, a reciprocal BLASTp comparison was run with an *e-value* equal to 1^e-6, considered a good hit for homology matches (Madden 2008). Results were visualized in Gephi, clustered with the force field and the Reingold–Watermann algorithm (Fig. 1). Graphical representation of the distribution of *pir* gene family and blast reciprocal genes between *P. gonderi* v2 and *P. cynomolgi* was performed with Circos software (Krzywinski et al. 2009).

### Orthologous Groups, GC Content, and Codon Usage

The following 26 reference genomes were used for these analyses: *P. adleri* G01 (Otto et al. 2018), *P. berghei* ANKA (Fougère et al. 2016), *P. billcolinsi* G01 (Otto et al. 2018), *P. blacklocki* G01 (Otto et al. 2018), *P. chabaudi* chabaudi (Otto et al. 2014), *P. coatneyi* Hackeri (Chien et al. 2016), *P. cynomolgi* M (Pasini et al. 2017), *P. falciparum* 3D7 (Böhme et al. 2018), *P. gaboni* SY75 (Otto et al. 2018), *P. gonderi* v2 (this study), *P. knowlesi* H (Pain et al. 2008), *P. malariae* UG01 (Rutledge et al. 2017), *P. ovale* curtisi G01 (Rutledge et al. 2017), *P. praefalciparum* G01 (Otto et al. 2018), *P. reichenowi* Centers for Disease Control and Prevention (CDC) (Otto et al. 2014), *P. relictum* SGS1-like (Böhme et al. 2018), *P. vivax* P01 (Auburn et al. 2016), *P. yoelii* yoelii 17 × (Otto et al. 2014), *Hepatocistys* sp. (Aunin et al. 2020), *P. fragile nilgiri* (GCA_000956335.1)*, P. gallinaceum* 8A (Böhme et al. 2018), *P. inui* San Antonio 1 (GCA_000524495.1), *P. malariae-like* GA01 (Rutledge et al. 2017), *P. ovale walkeri* CR01 (Rutledge et al. 2017), and *Plasmodium vinckei vinckei* (Ramaprasad et al. 2021) (supplementary table S11, Supplementary Material online).

Orthofinder2 software (Emms and Kelly 2019) was used with default parameters to identify orthogroups from 26 haemosporidian genomes, leading to 8,465 orthogroups. Of those, 1,961 orthogroups were SC-GO found in all the species, including those with limited genomic data (supplementary table S3, Supplementary Material online).

Each SC-OG was aligned at the protein level using Muscle v.3.8 (default parameters). Once aligned, they were reverse-translated with their respective nucleotide sequence as a template to obtain nucleotide alignments. The length of each SC-GO nucleotide alignment, excluding gaps, is reported in supplementary table S3, Supplementary Material online. In addition, all SC-GO alignments were concatenated to obtain an alignment of 2,498,205 bp (excluding gaps). Using SC-OGs allows comparing the species independently of their assembly quality (some are highly fragmented and have multiple scaffolds, such as the nonhuman primate parasites *Plasmodium fragile* and *Plasmodium inui*). However, all analyses were also carried out with all CDSs.

All CDSs from the 26 haemosporidian reference genomes were analyzed using the RSCU (Fig. 2, ssupplementary fig. S4 and table S4, Supplementary Material online), and the frequency of each amino acid (supplementary fig. S5 and table S5, Supplementary Material online). Both analyses were carried out using the Dambe6 software (Xia 2017). RSCU displays the difference between synonymous codon use and their actual usage. The ratio between the observed frequency of synonymous codons and the predicted frequency of that codon, when all codons are utilized without bias at that specific amino acid, was measured by RSCU. RSCU > 1 shows a preference for the codon, RSCU < 1 indicates that the codon is used less frequently than predicted, and RSCU = 1 indicates no bias in the codon usage (Goldman and Yang 1994).

### Analyzing Synteny Among *Plasmodium* Species

Due to their assembly quality, only 18 of the 26 genomes mentioned in the previous section were used for synteny-based analyses. However, to obtain more reliable results, problems associated with the assembly of *P. coatneyi* Hackeri were corrected (Chien et al. 2016). For the above, more than 300 million reads from 3 new Illumina libraries of the same strain were included in the data of Chien et al. (2016) (SRA accession number SAMN02595587 and SAMN04571750). The assembly followed the same methodology as *P. gonderi* v.2 (see above). The *de novo* structural annotation for this assembled genome did not differ from the available version, so RATT software (Otto et al. 2011) was used to transfer and update the annotation and gene order in the *P. coatneyi* genome. Orthofinder (see above) also detected the SC-OGs considered the “syntenic core genome” across the 18 genomes. The graphical representation of synteny was made using the R package GeneSpace (Lovell et al. 2022).

For synteny-based phylogenomic reconstruction, the pipeline of Zhao et al. (2021) (https://github.com/zhaotao1987/SynNet-Pipeline; Haug-Baltzell et al. 2017) was used, which includes 4 main steps: (i) Phylogenomic synteny network construction by reciprocal sequence similarity search of all 20 annotated parasitic proteomes using DIAMOND (version 0.9.14.115; default parameters; Buchfink et al. 2015), and pairwise synteny block detection by MCScanX36 (Wang et al. 2012) under these setting parameters b5s7m25 (b: number of top homologous pairs, s: number of minimum matched syntenic anchors, m: number of max gene gaps); this combination out of 27 (b: 5, 10, 15; s: 3, 5, 7; and m: 15, 25, 35) obtained the best result in terms of nodes and edges numbers (data not shown). (ii) Network clustering by R script available in Zhao's repository. (iii) Matrix representation, and (iv) maximum likelihood-based binary-tree inference in IQ-Tree (100 bootstrap replicates; Minh et al. 2020). Thus, the complete synteny network summarizes 729,572 pairwise synteny blocks and contains 92,498 nodes and 727,219 edges. Nodes are genes in synthetic blocks in the resulting synteny network, while edges connect synthetic anchor pairs (Fig. 3 and supplementary fig. S6, Supplementary Material online). As presented in the results (supplementary fig. S7, Supplementary Material online), the *P. vivax*-like Pv101 genome (Gilabert et al. 2018) was not used in the primary analyses due to the lack of several syntenic regions, but it was presented in Supplementary Materials.

Reconstruction of ancestral genomes across the *Plasmodium* genus was made using AGORA (Algorithm for Ancestral Gene Order Reconstruction; Muffato et al. 2023)). AGORA estimates all relevant pairwise gene order comparisons (avoiding gene gains, losses, and duplications). It assigns a weight to each possible gene-gene adjacency, corresponding to the number of pairwise comparisons that support this adjacency as ancestral at the node of interest. These weights are then used to solve a gene graph and extract the most likely gene order at the ancestor of interest. The ortholog groups were inferred for each internal node of the species tree using Orthofinder2 (see above).

Genes defined as essential genes characterized by Zhou et al. (2016) and “syntenic core genome” mapped to AGORA-defined syntenic blocks were used for GO enrichment analysis (Supek et al. 2011). These GO terms analyses were performed in PlasmoDB (The *Plasmodium* Genome Database Collaborative 2001) using *P. falciparum* 3D7 as a reference, with a *P*-value > 0.05 with Bonferroni correction (supplementary table S6 and table S7; supplementary supplementary table S7, figs. S8 and S9, Supplementary Material online).

### Phylogenomic Analyses

Phylogenetic relationships were inferred on each SC-GO nucleotide alignment using the ML method implemented in IQ-Tree (Minh et al. 2020) under the best-fit substitution model (supplementary table S3, Supplementary Material online), and 200 bootstrap replicates. Other parameters were set by default.

ASTRAL-III (Zhang et al. 2018a, 2018b) was employed to estimate a species tree under the multispecies coalescent model. It allows estimating an unrooted species tree given a set of unrooted gene trees; in this case, those gene trees were estimated with IQ-Tree (supplementary table S3, Supplementary Material online) from the SC-GOs. Normalized Robinson–Foulds distances between pairs of trees were calculated using the RF.dist function of the phangorn package in R with the parameter normalize = TRUE to characterize the degree of (mis)agreement between tree topologies (Schliep 2011).

Phylogenomic reconstructions were also inferred on a concatenated alignment with all the SC-GOs. This analysis was carried out using the Bayesian methods implemented in MrBayes v3.2.6 with the default priors (Ronquist and Huelsenbeck 2003) and the ML method in IQ-Tree (Minh et al. 2020). A general time-reversible model with gamma-distributed substitution rates and a proportion of invariant sites (GTR + Γ+I + G4) was used for both analyses, as it was determined using IQ-TREE, which searches for all nucleotide substitution models implemented in ModelFinder. The optimal model is selected based on its Bayesian Information Criterion (BIC) value.

Bayesian support for all nodes was inferred by sampling every 1,000 generations from 2 independent chains lasting 10^7^ Markov Chain Monte Carlo (MCMC) steps. The chains were assumed to have converged once the average standard deviation (SD) of the posterior probability was <0.01 and the value of the potential scale reduction factor (PSRF) was between 1.00 and 1.02 (Ronquist and Huelsenbeck 2003). As a “burn-in,” 50% of the sample was discarded once convergence was reached. In the case of ML analyses, 1,000 bootstrap replicates were evaluated for statistical confidence.

### Divergence Dating Analyses

Molecular clock analyses were performed using an alignment with 110 genes. These 110 genes were chosen based on the nRF distances between their topologies and the species tree. The threshold was 8 (or 4 changes between the gene tree and the species tree topologies; supplementary table S9, Supplementary Material online). Bayesian methods implemented in MCMCTree (Yang and Rannala 2006; Rannala and Yang 2007; Yang 2007) were used to estimate time trees and nucleotide substitution rates/My per partition. Two independent chains lasting 10^8^ MCMC steps were run for each analysis until convergence. Two scenarios were compared using a combination of calibration constraints. The calibration constraints include information on vertebrate hosts’ fossils (see Paleobiology Database at http://www.paleodb.org/). Although those are secondary calibrations, they are the only ones available, given the lack of *Plasmodium* fossils (Pacheco and Escalante 2023). In all cases, uniform priors were used to calibrate divergences. No particular time point was favored within a given time interval. No punctual calibrations were used.

The first calibration constraint considered that *P. gonderi* found in *Mandrillus* spp. and *Cercocebus* spp. diverged from those *Plasmodium* species found in Southeast Asia macaques when *Macaca* branched from *Papio* (Mu et al. 2005; Pacheco et al. 2011). *Macaca* spp. fossils indicate that such events took place 6 to 8 MYA as minimum and maximum boundaries, respectively (Delson 1980). However, these fossils are used as minimum time boundaries when studying primates (Perelman et al. 2011). Thus, a more inclusive alternative calibration constraint was proposed around this event, assuming that the divergence at the same node occurred between 6 and 14.2 MYA. Thus, this interval was used as a uniform prior (no particular time point was favored). This inclusive time interval incorporates molecular estimates for the same *Papio*-*Macaca* divergence event (Pacheco et al. 2011), and is also consistent with older fossils reported for *Macaca* spp. (see Paleobiology Database at http://www.paleodb.org/), and considers that *P. gonderi* has also been reported in *Chlorocebus thuscopithecini*.

A second fossil-based calibration constraint is the minimum of 23.5 MYA for the human/*Macaca* split (Benton and Donoghue 2007), which is assumed to be the minimum time when the monophyletic group, including *P. malariae* (a parasite found in humans) and all the Asian parasites, originated. This event is used to inform a calibration prior with a maximum of 65 MYA that allows this parasite clade to be as old as the origin of primates if the data support that (Perelman et al. 2011, Ksepka et al. 2015).

These calibrations were organized into 2 scenarios. Scenario 1 considers only the relaxed 6 to 14.2 MYA calibration of the *Papio*/*Macaca* parasite divergence (95% quantile = 13.8) (Delson 1980; Mu et al. 2005). The second scenario included the previous calibration and the one with a minimum of 23.5 and a maximum of 34.0 MYA for the human/*Macaca* split (95% quantile = 33.5). A third scenario was added to explore the effect of calibration in the clade with higher AT content, *Laverania*. In particular, the origin of the *Laverania* subgenus, a monophyletic group of parasites found in African Apes, was assumed to be the divergence of all the extant hosts of these parasites (Homininae that includes *Homo-Pan-Gorilla*) with a maximum in the Hominidae (Great Apes including *Pongo*). The calibration was uniform, with a minimum of 6.5 MYA and a maximum of 15.2 MYA.

The MCMC algorithm was used to estimate divergence times on the constrained tree topology in 2 separate runs using the approximate likelihood calculation (dos Reis and Yang 2011). We used baseml under the strict clock model to drive a prior for the overall substitution rate G(1.2,1.0). Given the lack of prior information, both independent and autocorrelated rate models (Rannala and Yang 2007) were employed with the following default parameters: a rate drift parameter σ2 G(1,1); and the parameters of the birth-death process with species sampling fixed at λ = μ = 1 and ρ = 0. The chains were run until effective sample size (ESS) values were higher than 200 after discarding the burn-in, sampling every 1000th cycle. As MCMCTree requires some prior information at the root age, a loose maximum of 86.8 MYA was provided to limit the age of the most recent common ancestor of *Plasmodium*. All the calibrations were uniform priors with soft bounds.

Besides the Bayesian inferences, divergence times were also estimated in a non-Bayesian framework with the RelTime method (Tamura et al. 2012). RelTime algorithm does not assume any statistical model for the variation in rates of evolution across lineages as the Bayesian methods do (Tamura et al. 2012). Although it estimates relative times, it is not free from assumptions. RelTime computes the rate of the ancestral branch as the average of those in the 2 immediate descendant branches (Tamura et al. 2018). In this case, calculations were carried out on the command-line version of MEGA11 (Tamura et al. 2021). The substitution model was the same as for Bayesian analyses (GTR+Γ+I). Calibration information was provided as uniform distributions in RelTime, using the same minimum and maximum boundaries used in MCMCTree. As RelTime does not allow calibrations to be placed at the root of the phylogenetic tree (Tamura et al. 2021), we used a maximum of 86.8 MYA at the root of the ingroup clade. The same calibration scenarios employed in MCMCTree were used to infer divergence times in RelTime.

## Supplementary Material

evae027_Supplementary_Data

## Data Availability

Scripts used to perform the analyses reported in this article are available at https://github.com/EscalanteLab/Plasmodium-gonderi-genome-analysis. The data used in this work is available under the BioProject GenBank accession no. PRJNA928704 and the Genome Whole Sequence GenBank accession no. JARBEB000000000.1.
