# Correction to: The Genome of *Plasmodium gonderi*: Insights into the Evolution of Human Malaria Parasites

**DOI:** 10.1093/gbe/evae057

**Published:** 2024-03-25

This is a correction to: Axl S Cepeda, Beatriz Mello, M Andreína Pacheco, Zunping Luo, Steven A Sullivan, Jane M Carlton, Ananias A Escalante, The Genome of *Plasmodium gonderi*: Insights into the Evolution of Human Malaria Parasites, *Genome Biology and Evolution*, Volume 16, Issue 2, February 2024, evae027, https://doi.org/10.1093/gbe/evae027

In the originally published version of this article, the file containing Supplementary figures S1- S12 was inadvertently omitted from the Supplementary data section.

This has been corrected.

