# Supplementary material for: The Genome of Plasmodium gonderi: Insights into the Evolution of Human Malaria Parasites
Source: Genome Biol Evol. 2024 Feb 20;16(2):evae027. doi: 10.1093/gbe/evae027 (PMC10901558; doi:10.1093/gbe/evae027)

Supplementary Material

Article (Discoveries)

The Genome of *Plasmodium gonderi*: Insights into the Evolution of Human Malaria Parasites.

Axl Cepeda S[1], Beatriz Mello[2], M. Andreína Pacheco[1], Zunping Luo[3], Steven A Sullivan[3], Jane M. Carlton[3], Ananias A. Escalante[1*].

[1]Biology Department/Institute of Genomics and Evolutionary Medicine (iGEM), Temple University, Philadelphia, Pennsylvania 19122-1801, USA.

[2]Departamento de Genética, Universidade Federal do Rio de Janeiro, Rio de Janeiro, Brazil

[3]Center for Genomics & Systems Biology, Department of Biology, New York University, 12 Waverly Place, New York, NY, 10003, USA

Supplementary figure S1. Graphical representation of *P. gonderi* v2 genome. The map was designed with Circos software (Krzywinski et al. 2009). From the outside to the center: Karyotype; distribution of genes (in blue strand 5'-3' and orange strand 3'-5'); AT content: 2500bp window size. The lower blue border means 50% of the total nucleotide content, and the dark gray line shows the average AT content of the total genome (73.52%). Values above and below this mean are represented in green or red, respectively; coverage: 2500bp window size. The red and gray lines represent 500X and 4500X coverage, respectively.

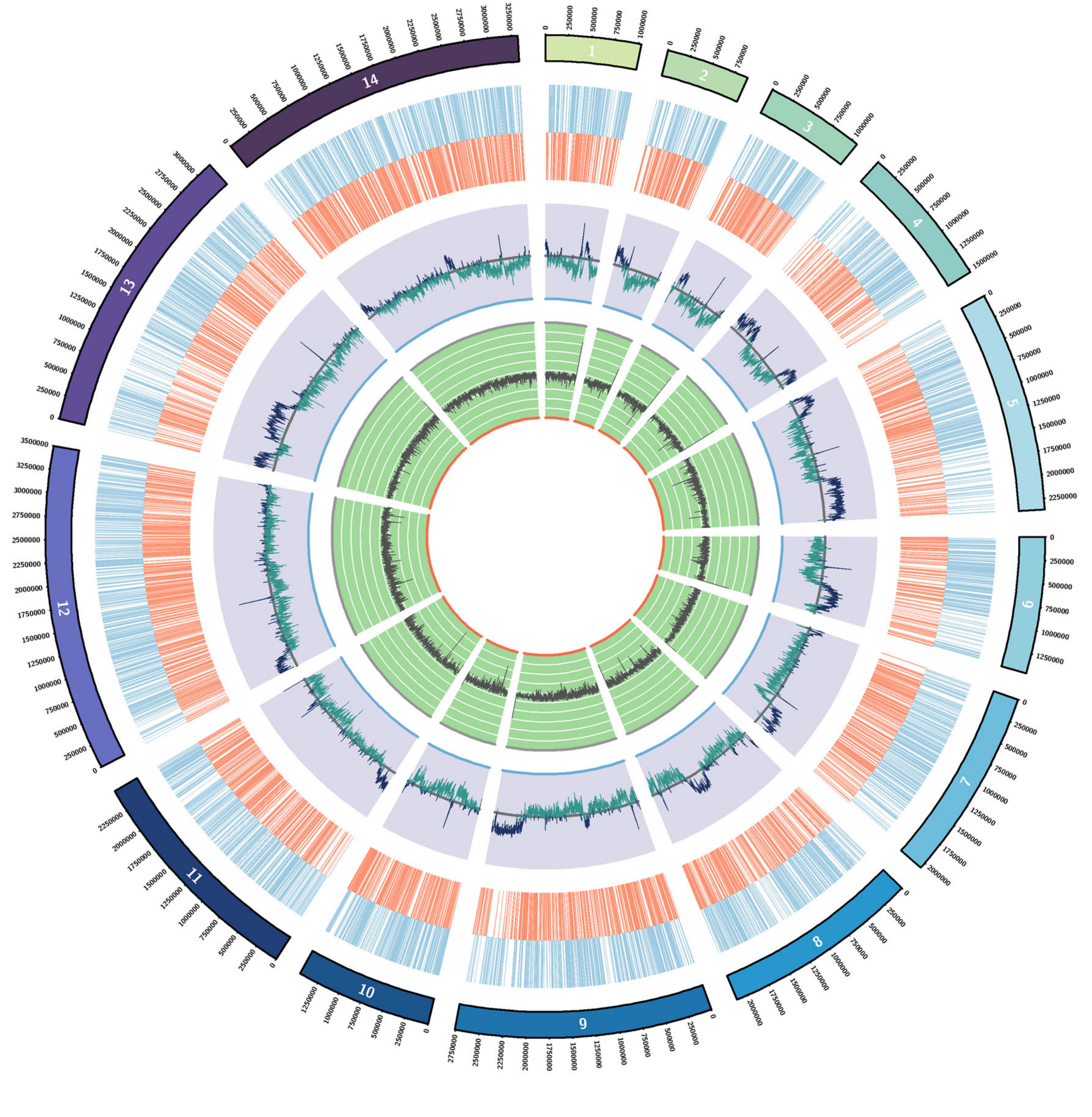

Supplementary figure S2. Distribution of *pir* genes across chromosomes in *P. gonderi* v1, and *P. gonderi* v2. In blue, those genes are on 5'-3' strand, and in green, genes are on 3'-5' strand.

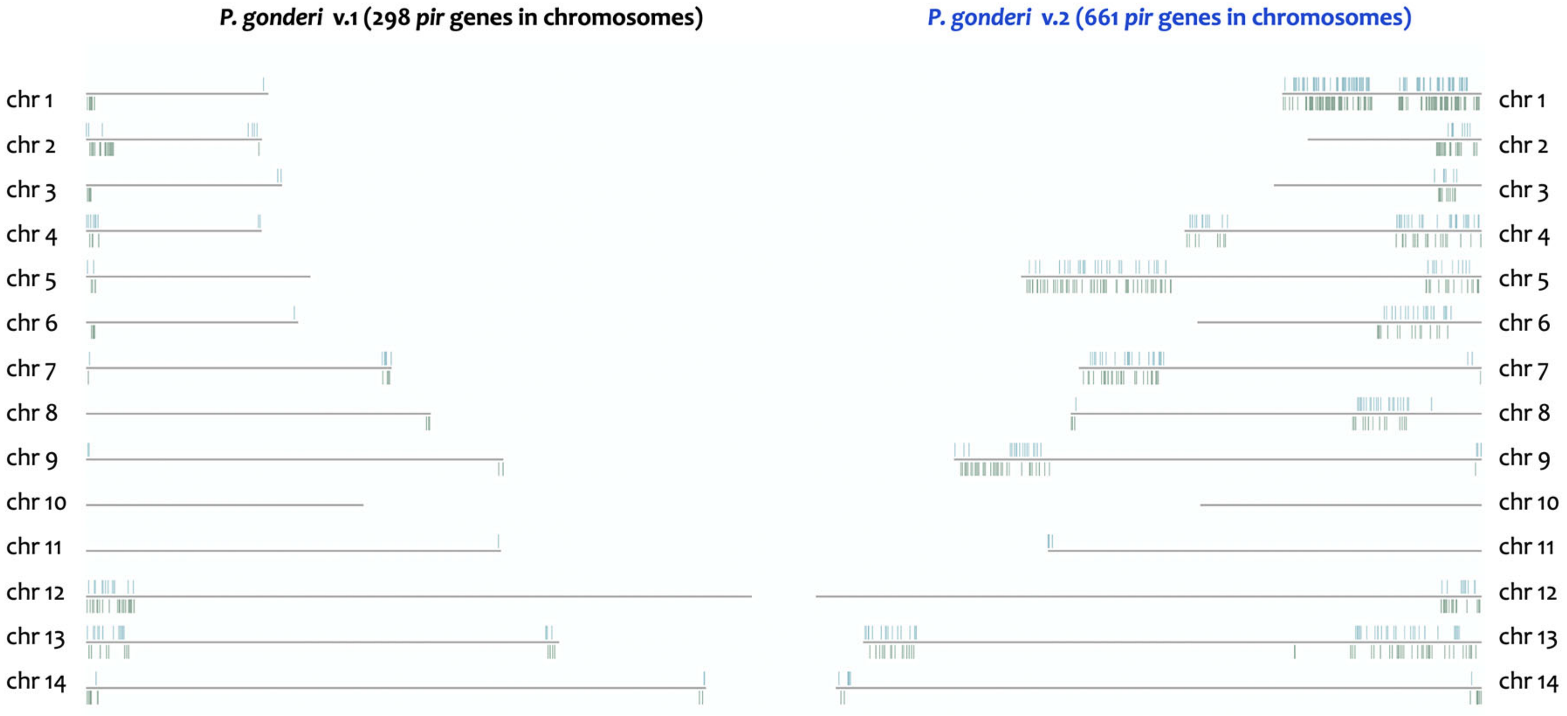

Supplementary figure S3. Graphical representation of the synteny between *P. gonderi v2* and *P. cynomolgi*. From the outside to the center: Karyotype of both genomes (in purple *P. gonderi* and green *P. cynomolgi*). The blue ribbon shows the orthologous sequences between the genomes. The orange ribbon represents the orthology between the *pir* genes including genes not located in chromosomes in both genomes. The orthology analysis was performed using Orthofinder software (Emms and Kelly 2019).

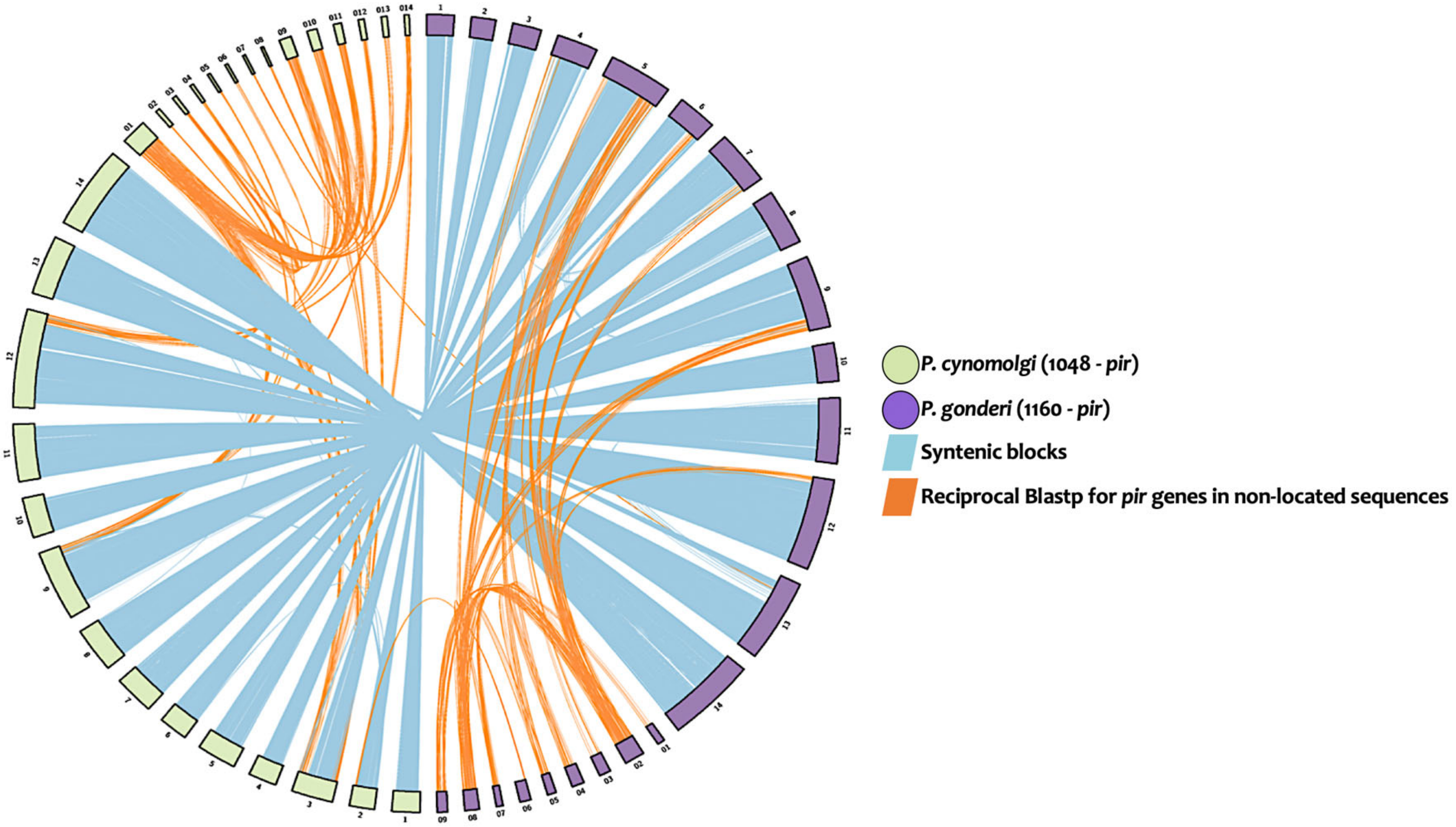

Supplementary figure S4. Hierarchical cluster by average and heat map of Relative Synonymous Codon Usage (RSCU) values of each codon in all the CDS found in haemosporidian genomes regardless if there were orthologs among all species. Each square in the heat map represents the RSCU frequency value of each codon (in rows) within the CDS of each genome (in columns). Colors indicate the magnitude of RSCU values.

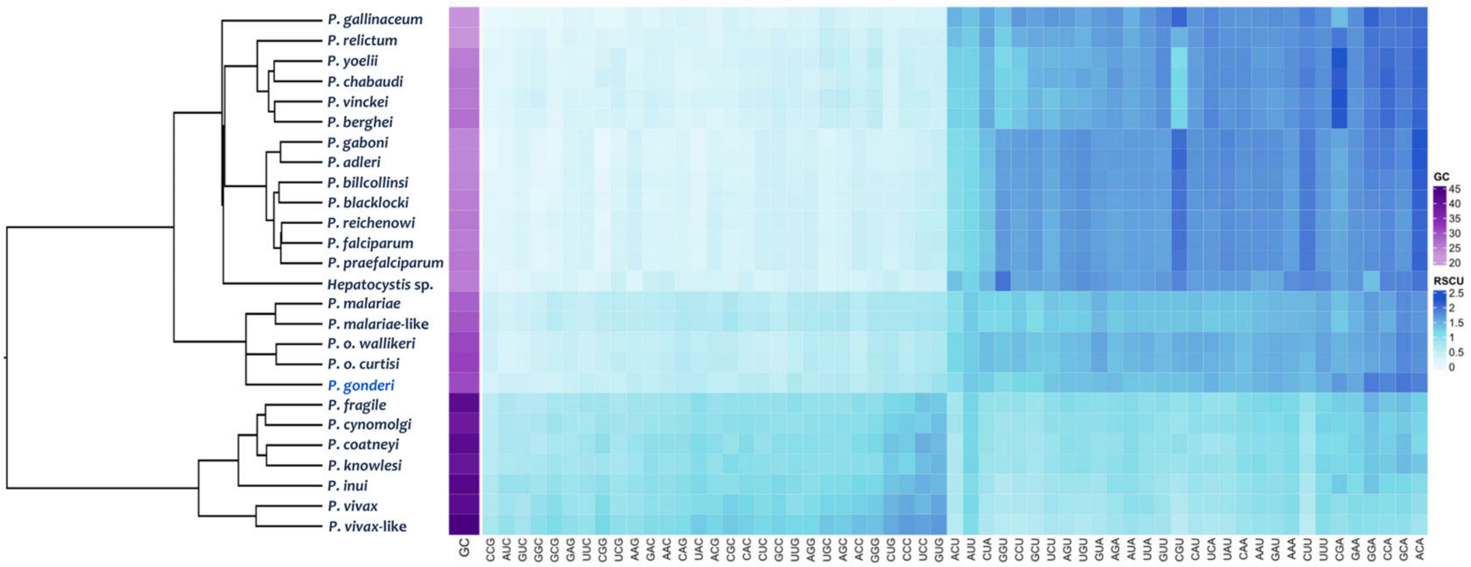

Supplementary figure S5. Hierarchical cluster by average and heat map of each codon's amino acid frequency values in the SC-OG and CDS of Haemosporida genomes. Each square in the heat map represents the amino acid frequency value (in rows) within the SC-OG or CDS of each Haemosporida genome (in columns). Colors indicate the magnitude of amino acid frequency values.

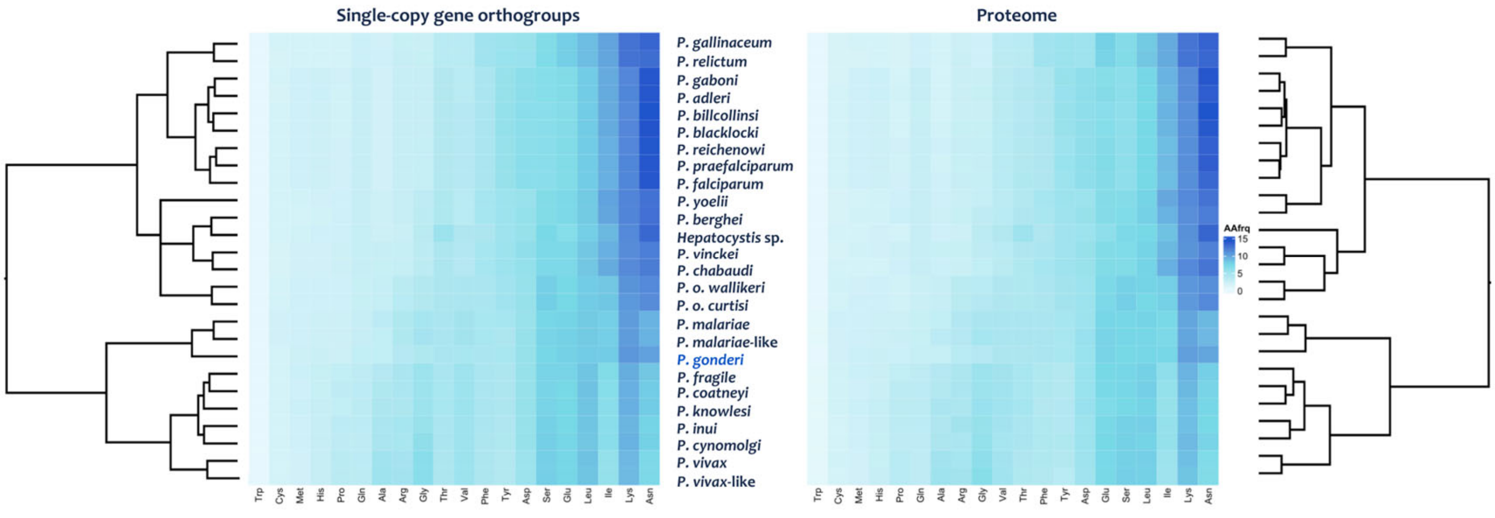

Supplementary figure S6. Phylogenomic reconstruction based on synteny and graphical representation of the syntenic blocks across *Plasmodium* genus using the currently available assembly of *P. coatneyi* Hackeri. There were changes in syntenic patterns likely due to assembly problems. However, the overall phylogenetic reconstruction is robust compared to fig. 3. Phylogenetic reconstruction was inferred by Maximum Likelihood methods implemented in IQ-tree software (100 bootstrap replicates) from the synteny matrix obtained following the protocol of Zhao et al. (2021). The synthetic blocks were graphed with the R package GeneSpace (Lovell et al. 2022).

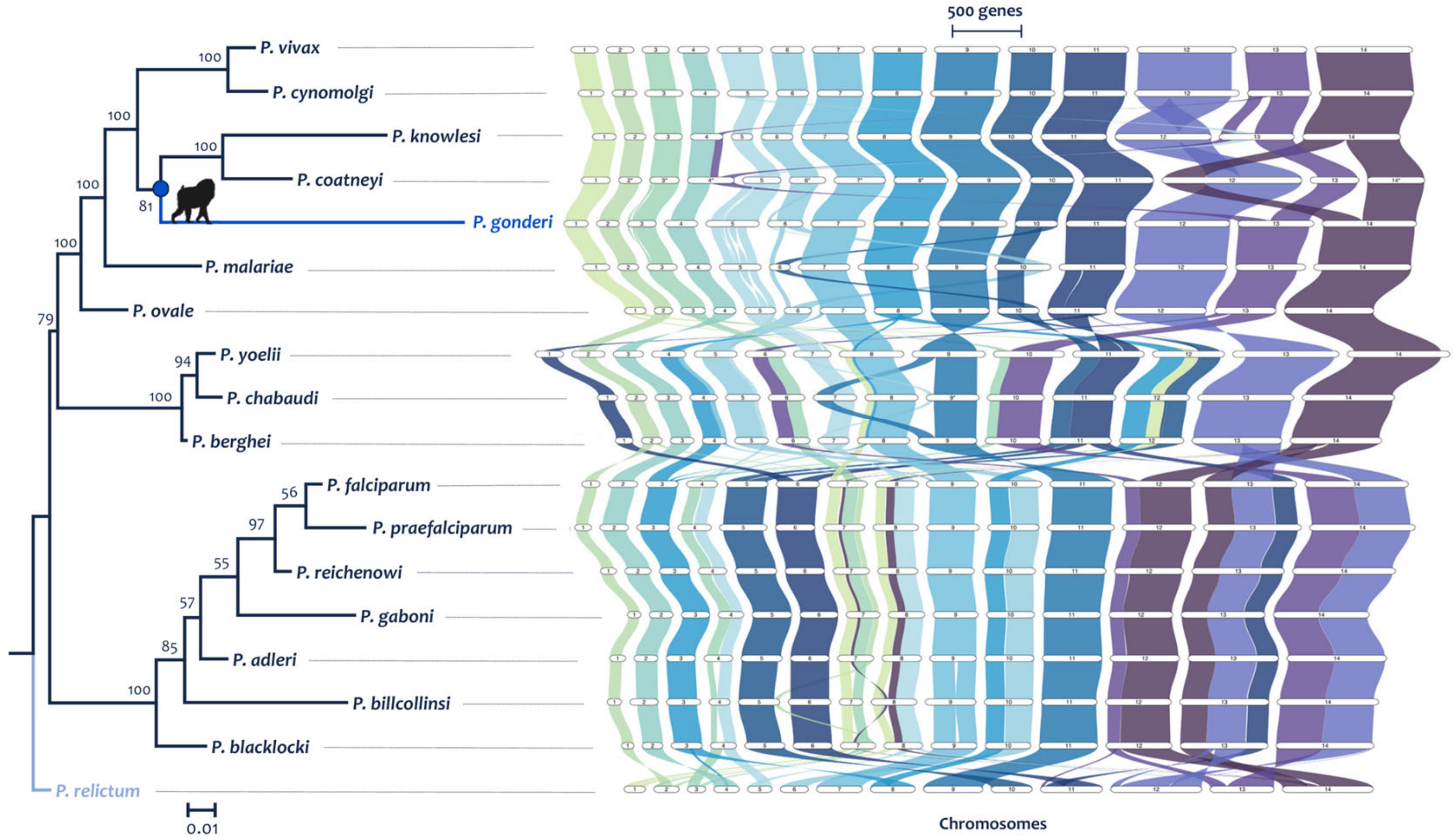

Supplementary figure S7. Hierarchical clustering by average and heatmap of syntenic blocks of *Plasmodium* reference genomes. Each square of the heatmap represents the presence (in blue) or absence (in white) of the syntenic block by species. The cluster shows the incompleteness of the *P. vivax*-like.

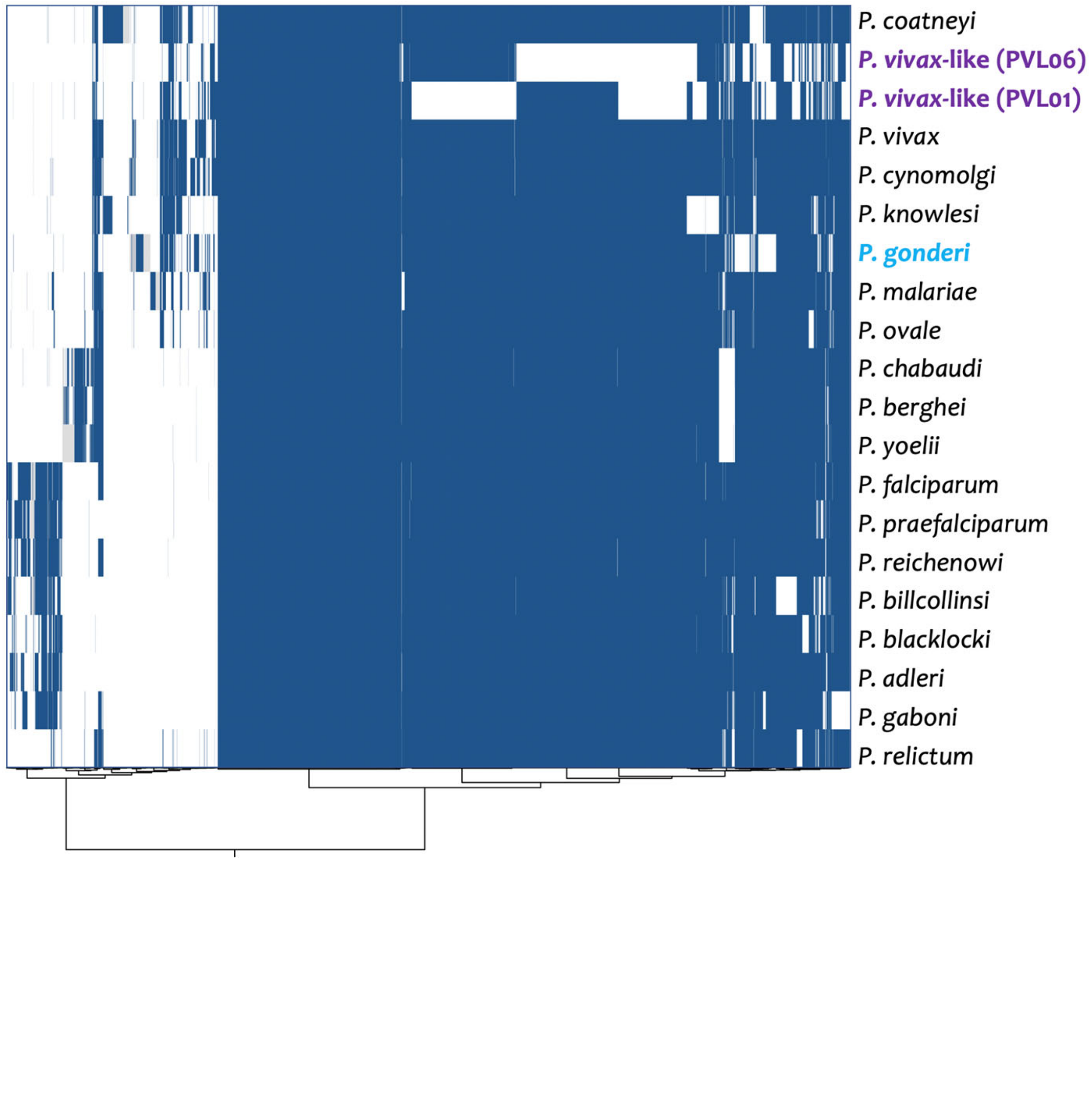

Supplementary figure S8. Gene ontology enrichment. GO terms were searched in PlasmoDB using each *P. falciparum* gene ID of putatively essential genes as defined by Zhang et al. (2018) located at the A) syntenic blocks, and B) the breakpoints for the common ancestor of all *Plasmodium*, the common ancestor of mammalian *Plasmodium*, and the common ancestor of *Laverania* subgenus reconstructed with AGORA (Muffato et al 2023).

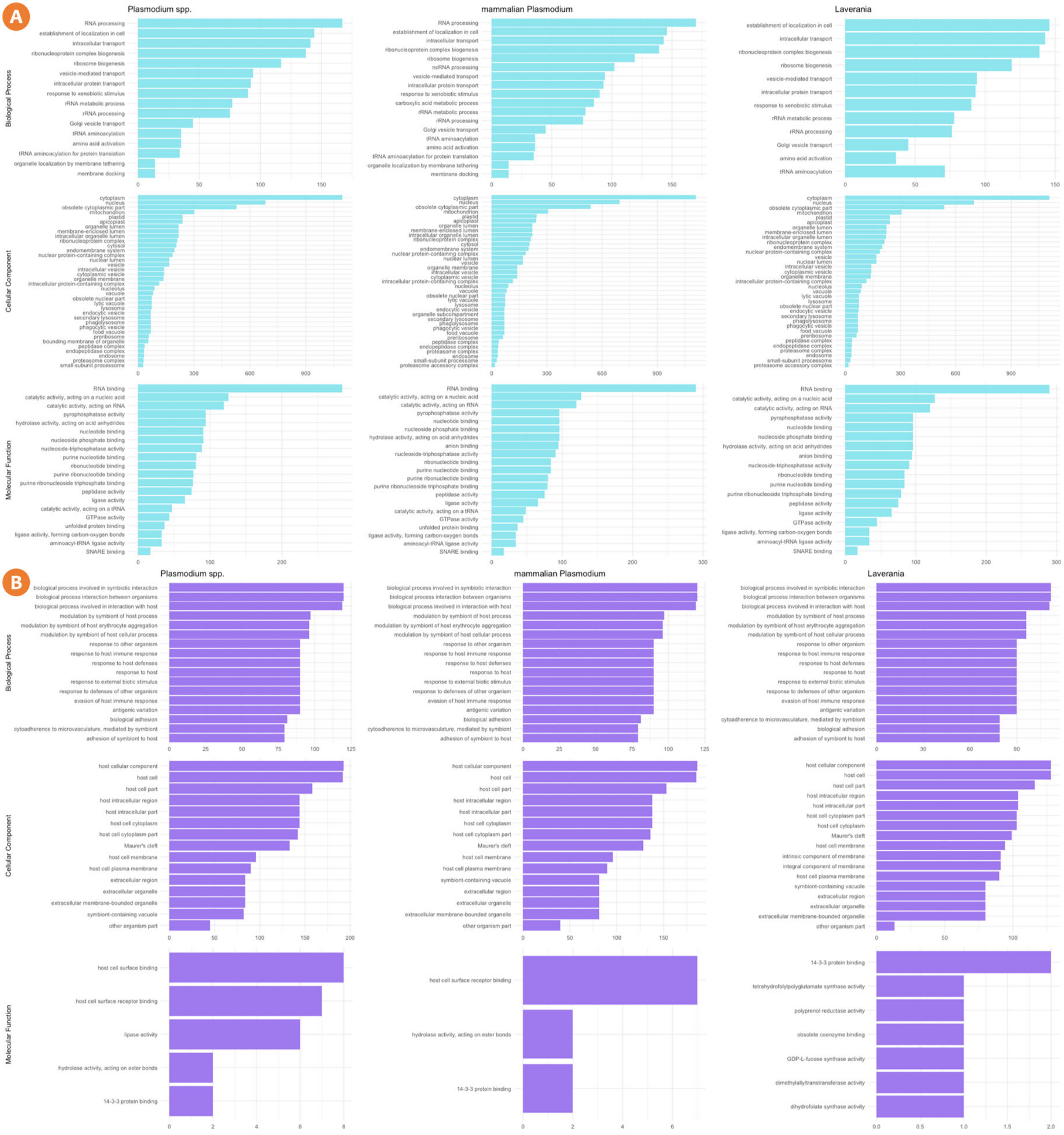

Supplementary figure S9. Gene Ontology Enrichment of the 3,408 SC-OGs or "core genome," syntenic "no-core," and breakpoints-"no-core" as defined by the 18 *Plasmodium* reference genomes. The GO terms were searched in PlasmoDB using the ID of each *P. falciparum* gene; subsequently, these GO terms were plotted using R packages.

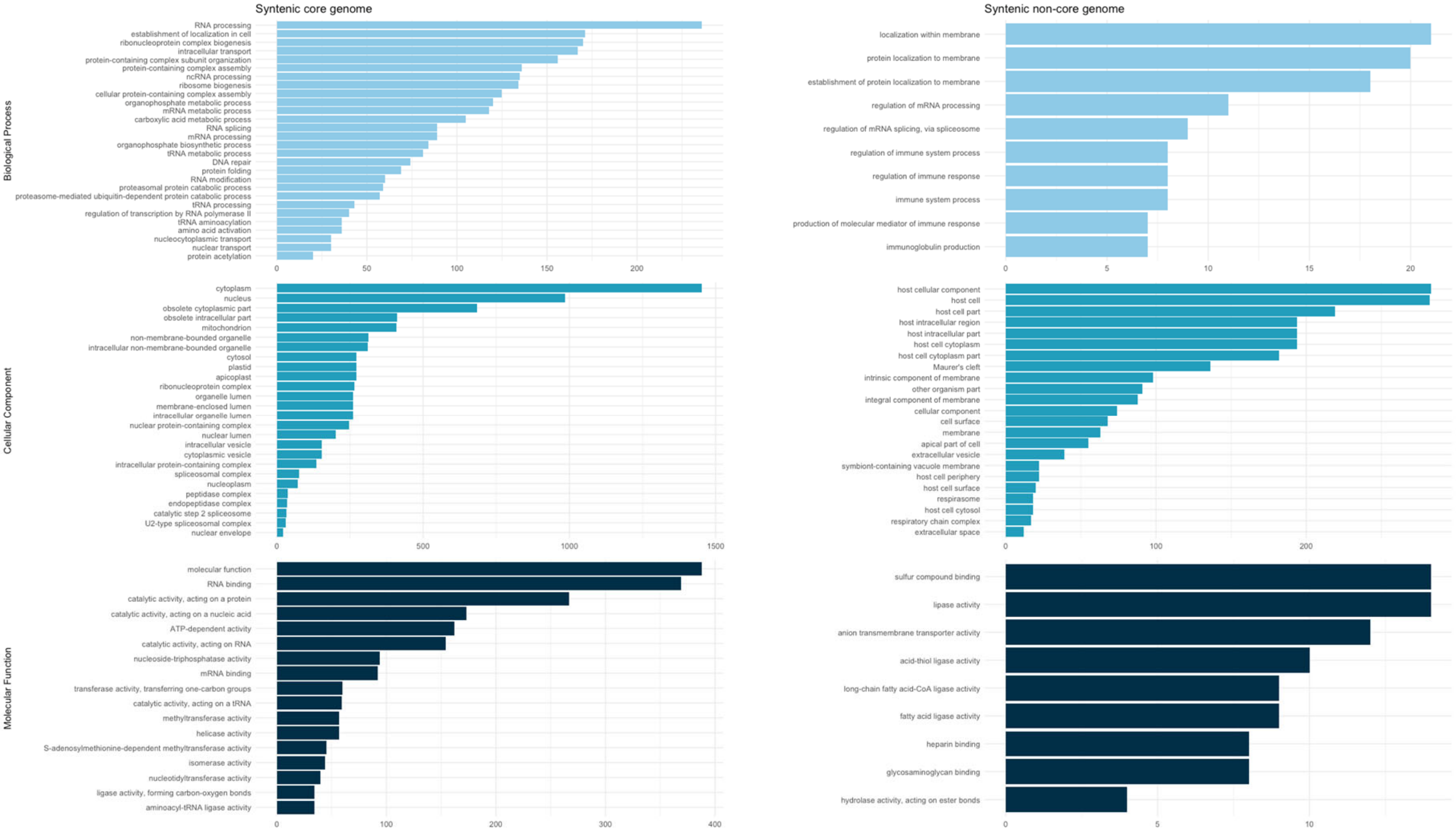

Supplementary figure S10. Labels on the nodes from the phylogeny used in the molecular dating analyses.

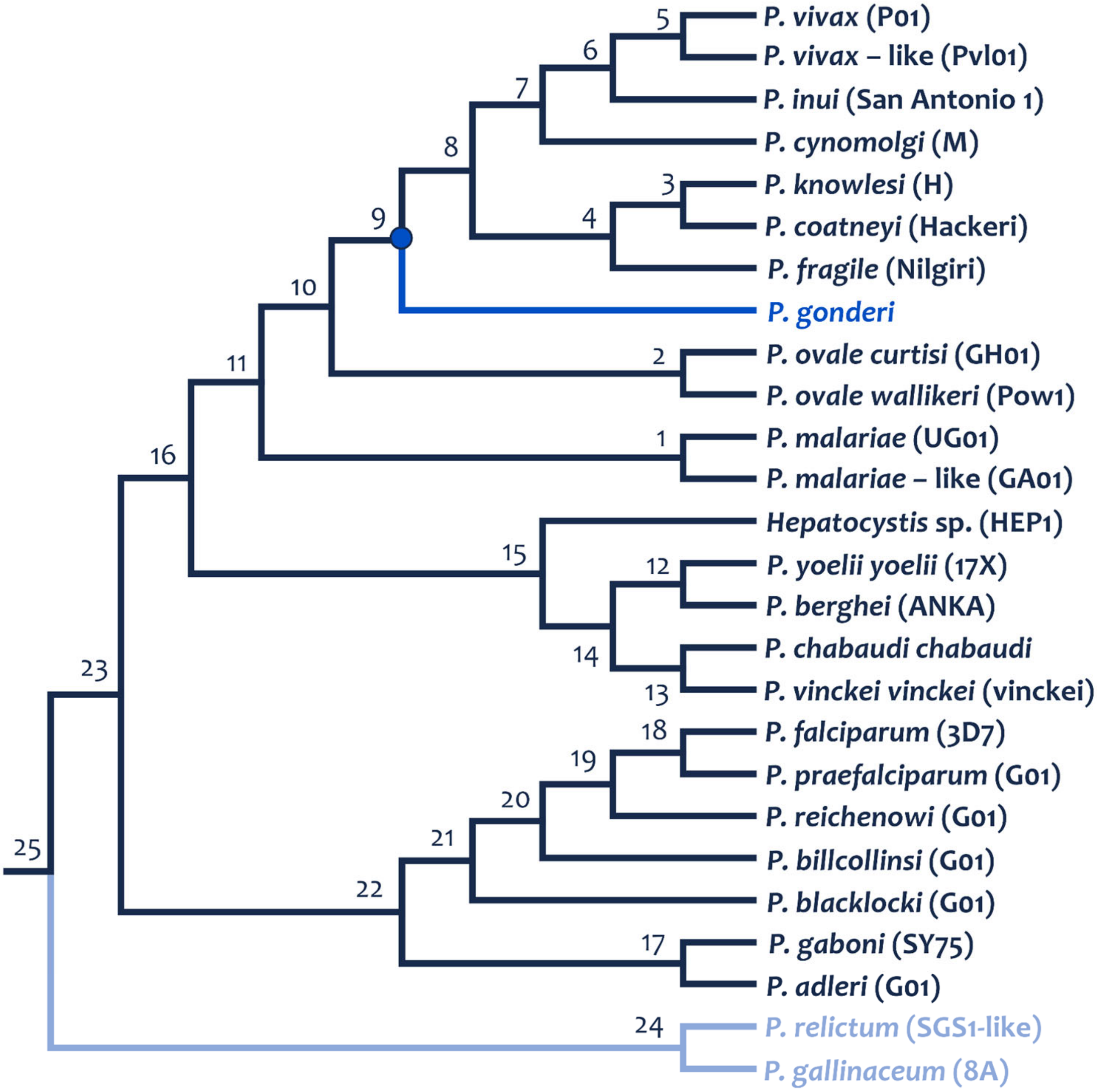

Supplementary figure S11. Regression analyses between the divergence times estimated in MCMCtree and RealTime under distinct rate models and calibration scenarios.

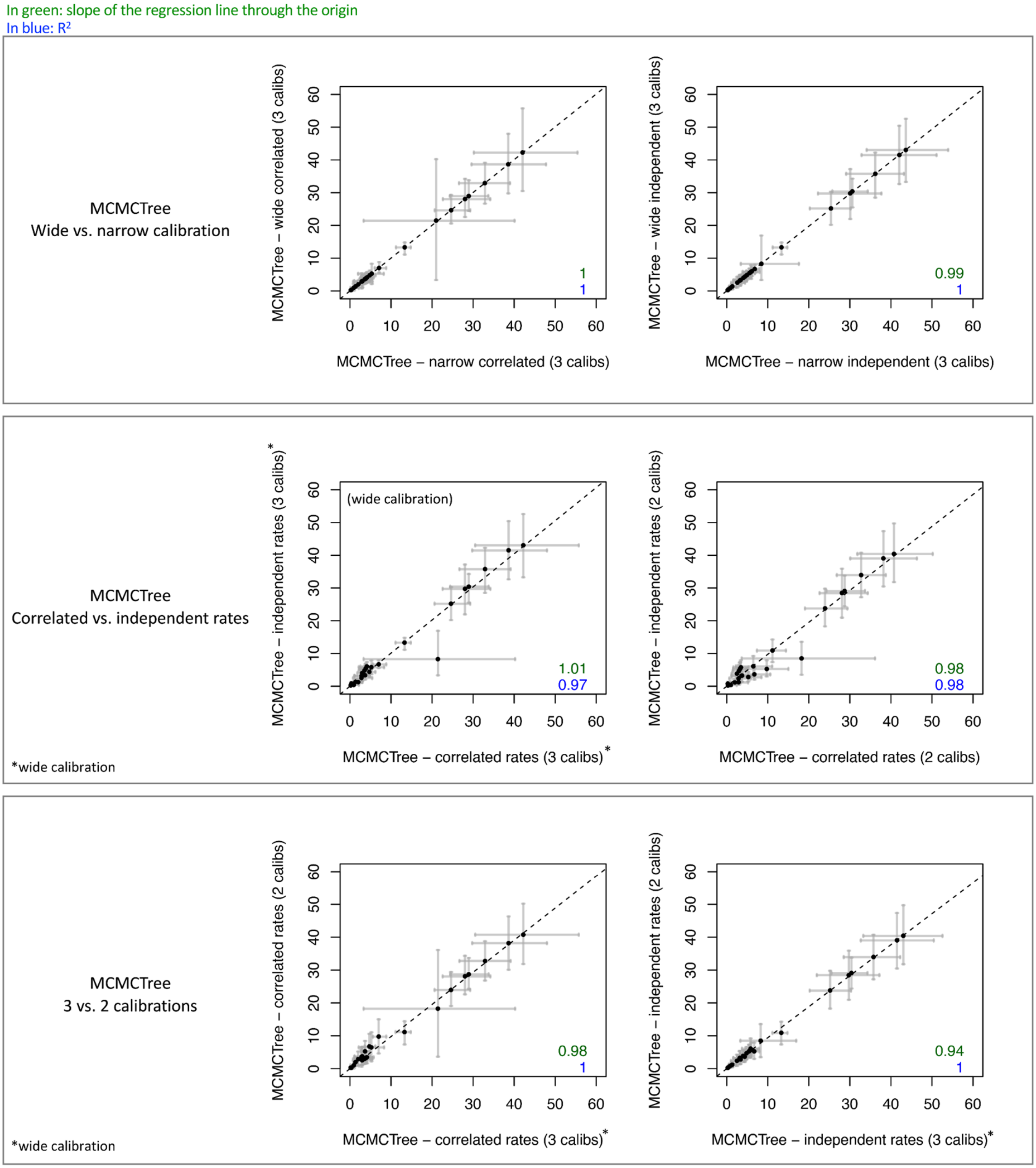

Supplementary figure S11…

In green: slope of the regression line through the origin
In blue: R^2

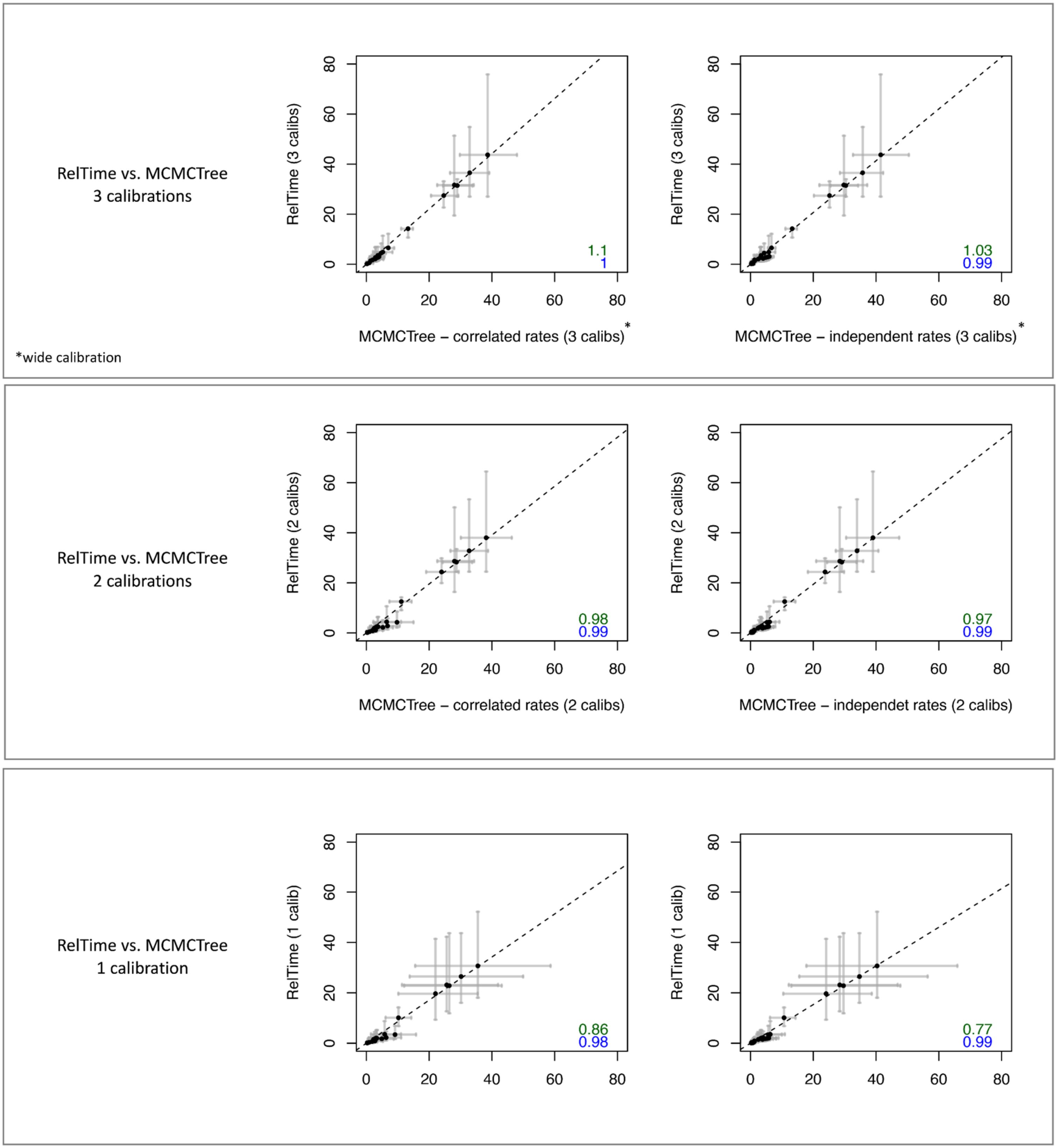

Supplementary figure S11…

In green: slope of the regression line through the origin
In blue: R^2

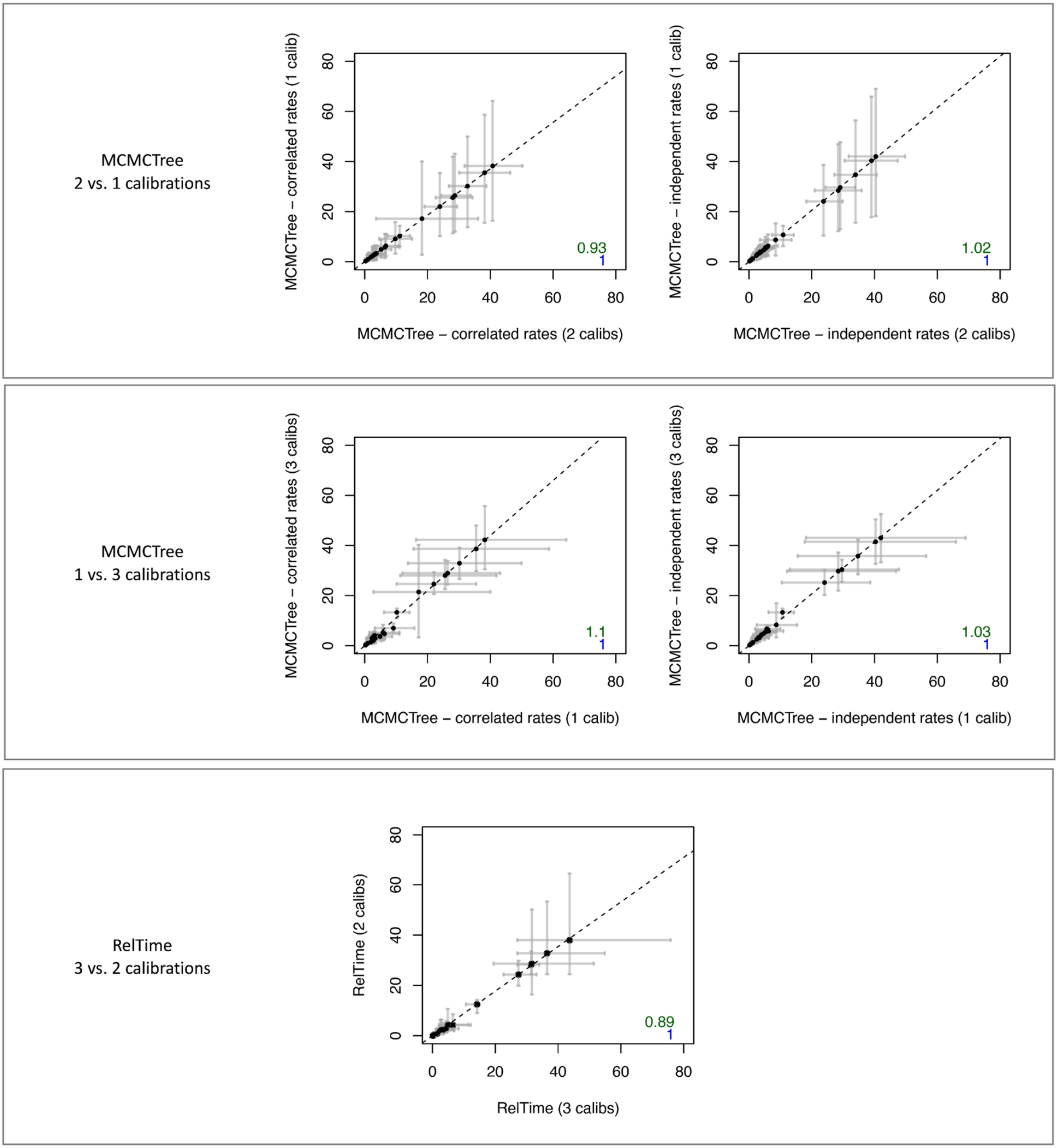

Supplementary figure S12. Time tree of the primate malarias using 110 genes. Divergence times were estimated using MCMCTree under the (A) autocorrelated and (B) independent rate models, using only two calibration constraints. Calibrations were uniform priors, as explained in the text. Times are shown in MYA. 95% CrIs for the major clades are shown in parentheses next to the nodes.

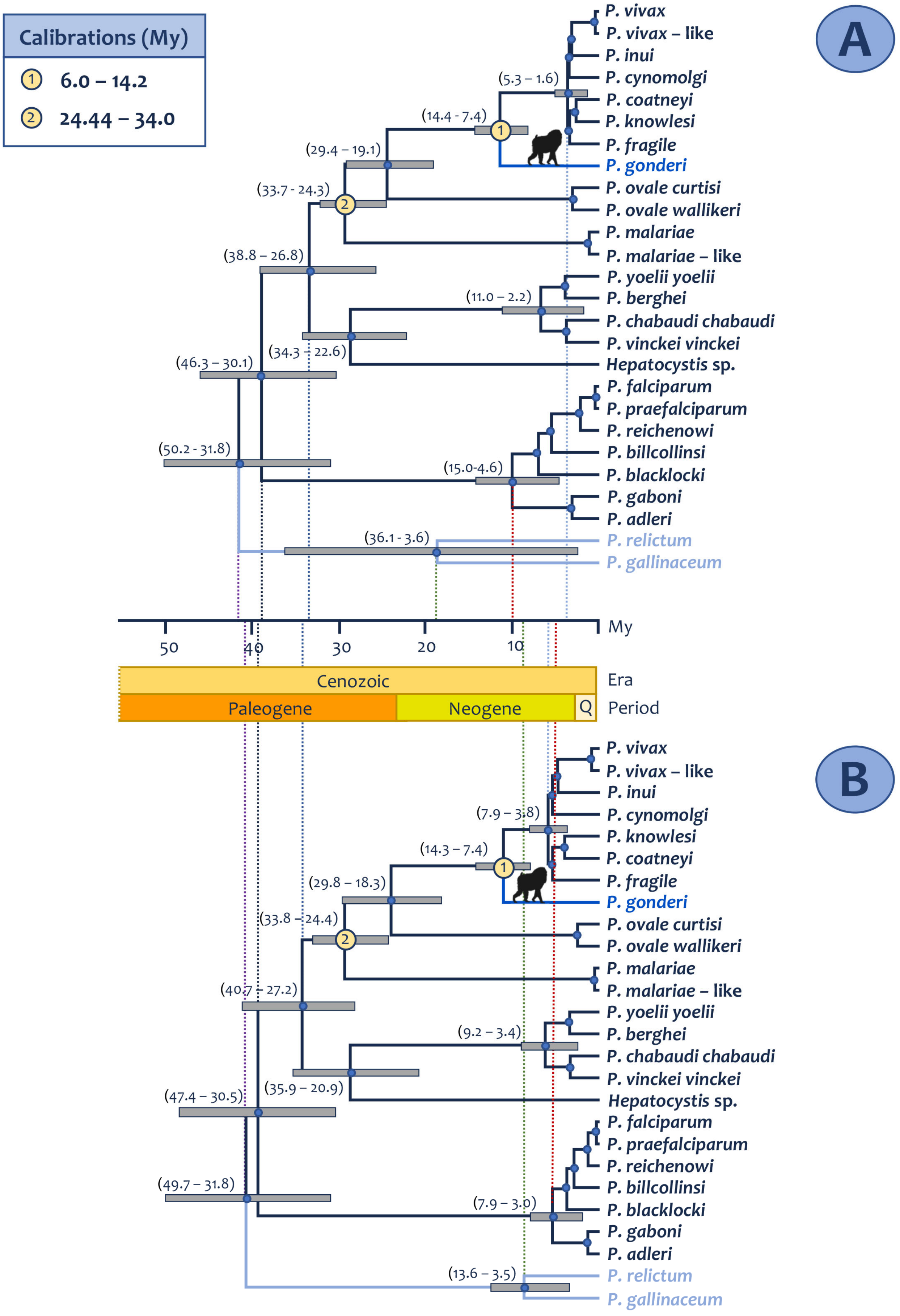

Supplement: evae027_Supplementary_Data [file evae027_supplementary_data.zip › Supplementary material_March2024.pdf]
